# Supplementary material for: Mapping the distribution of sandflies and sandfly-associated pathogens in China
Source: PLoS Negl Trop Dis. 2024 Jul 16;18(7):e0012291. doi: 10.1371/journal.pntd.0012291 (PMC11251628; doi:10.1371/journal.pntd.0012291)
Supplement: S1 Appendix — Text A. Data Collection and Management. Text B. Ecological modeling and clustering analysis of sandflies. Text C. Ecological Modeling of VL. Fig A. The spatial distribution of the 673 counties with at least one record of sandflies (yellow) from 1940 to 2022, China. Base layers of the maps were downloaded from Resource and Environment Science and Data Center (https://www.resdc.cn/DOI/DOI.aspx?DOIID=120). Fig B. The number of records related to sandflies in different provinces in different periods (with the number of publications in parentheses) and a comparison of the total number of records for four VL vector sandflies in each province. Fig C. The spatial distribution of the sandfly genus Phlebotomus recorded at the county level from 1940 to 2022 in China. Base layers of the maps were downloaded from Resource and Environment Science and Data Center (https://www.resdc.cn/DOI/DOI.aspx?DOIID=120). Fig D. The spatial distribution of the sandfly genus Sergentomyia recorded at the county level from 1940 to 2022 in China. Base layers of the maps were downloaded from Resource and Environment Science and Data Center (https://www.resdc.cn/DOI/DOI.aspx?DOIID=120). Fig E. The spatial distribution of the sandfly genus Chinius and Idiophlebotomus recorded at the county level from 1940 to 2022 in China. Base layers of the maps were downloaded from Resource and Environment Science and Data Center (https://www.resdc.cn/DOI/DOI.aspx?DOIID=120). Fig F. Sandfly species richness (circles) at the prefecture level in seven biogeographic zones in the mainland of China from 1940 to 2022. Base layers of the maps were downloaded from Resource and Environment Science and Data Center (https://www.resdc.cn/DOI/DOI.aspx?DOIID=120). Fig G. The predicted county-level distributions of the six most prevalent sandfly species in the Phlebotomus genus, averaged over the ensemble of BRT models. Base layers of the maps were downloaded from Resource and Environment Science and Data Center (https://www.resd [file pntd.0012291.s001.pdf]

S1 Appendix

Supplement to: Mapping the distribution of sandflies and sandfly-associated pathogens in China

Table of Contents

Table of Contents......1
Supplementary Materials and Methods......3
Text A: Data Collection and Management......3
Text B: Ecological modeling and clustering analysis of sandflies......4
Text C: Ecological Modeling of VL......5
Supplement References 1......6
Supplementary Figures......7
Fig A: The spatial distribution of the 673 counties with at least one record of sandflies (yellow) from 1940 to 2022, China. Base layers of the maps were downloaded from Resource and Environment Science and Data Center (https://www.resdc.cn/DOI/DOI.aspx?DOIID=120). 7
Fig B: The number of records related to sandflies in different provinces in different periods (with the number of publications in parentheses) and a comparison of the total number of records for four VL vector sandflies in each province. 8
Fig C: The spatial distribution of the sandfly genus Phlebotomus recorded at the county level from 1940 to 2022 in China. Base layers of the maps were downloaded from Resource and Environment Science and Data Center (https://www.resdc.cn/DOI/DOI.aspx?DOIID=120). 9
Fig D: The spatial distribution of the sandfly genus *Sergentomyia* recorded at the county level from 1940 to 2022 in China. Base layers of the maps were downloaded from Resource and Environment Science and Data Center (https://www.resdc.cn/DOI/DOI.aspx?DOIID=120). 10
Fig E: The spatial distribution of the sandfly genus *Chinius* and *Idiophlebotomus* recorded at the county level from 1940 to 2022 in China. Base layers of the maps were downloaded from Resource and Environment Science and Data Center (https://www.resdc.cn/DOI/DOI.aspx?DOIID=120). 11
Fig F: Sandfly species richness (circles) at the prefecture level in seven biogeographic zones in the mainland of China from 1940 to 2022. Base layers of the maps were downloaded from Resource and Environment Science and Data Center (https://www.resdc.cn/DOI/DOI.aspx?DOIID=120)..12
Fig G: The predicted county-level distributions of the six most prevalent sandfly species in the *Phlebotomus* genus, averaged over the ensemble of BRT models. Base layers of the maps were downloaded from Resource and Environment Science and Data Center (https://www.resdc.cn/DOI/DOI.aspx?DOIID=120). 13
Fig H: The predicted county-level distributions of the six most prevalent sandfly species in the *Sergentomyia* genus, averaged over the ensemble of BRT models. Base layers of the maps were downloaded from Resource and Environment Science and Data Center (https://www.resdc.cn/DOI/DOI.aspx?DOIID=120). 14
Fig I: The mean curves (red) and 95% percentiles (gray) for the effects of major predictors (RC ≥5%) on the probability of occurrence of 12 main sandfly species based on the ensemble of BRT models. 15
Fig J: XGBoost-model-predicted AVL and DT-ZVL incidence in response to major predictors (RC ≥5%) when other predictors are fixed at mean values. 17
Fig K: XGBoost-model-predicted MT-ZVL incidence in response to major predictors (RC ≥5%) when other predictors are fixed at mean values. 18
Fig L: Spatial distribution and changes in model-predicted incidence of VL in the mainland of China under SSP126. Base layers of the maps were downloaded from Resource and Environment Science and Data Center (https://www.resdc.cn/DOI/DOI.aspx?DOIID=120). 19
Fig M: Spatial distribution and changes in model-predicted incidence of VL in the mainland of China under SSP245. Base layers of the maps were downloaded from Resource and Environment Science and Data Center (https://www.resdc.cn/DOI/DOI.aspx?DOIID=120). 20
Fig N: Spatial distribution and changes in model-predicted environmental suitability of *P. wui* in future under three scenarios. Base layers of the maps were downloaded from Resource and Environment Science and Data Center (https://www.resdc.cn/DOI/DOI.aspx?DOIID=120). 21
Fig O: Spatial distribution and changes in model-predicted environmental suitability of *P. chinensis* in future under three scenarios. Base layers of the maps were downloaded from

Resource and Environment Science and Data Center (https://www.resdc.cn/DOI/DOI.aspx?DOIID=120). 22
Fig P: Spatial distribution and changes in model-predicted environmental suitability of *P. longiductus* in future under three scenarios. Base layers of the maps were downloaded from Resource and Environment Science and Data Center (https://www.resdc.cn/DOI/DOI.aspx?DOIID=120). 23
Fig Q: Spatial distribution and changes in model-predicted environmental suitability of *P. alexandri* in future under three scenarios. Base layers of the maps were downloaded from Resource and Environment Science and Data Center (https://www.resdc.cn/DOI/DOI.aspx?DOIID=120). 24
Supplementary Tables...25
Table A: The specific references for all 47 sandfly species in China from 1940 to 2022. 25
Table B: The inclusion and exclusion criteria for screening publications.......................................27
Table C: Original resolutions and extents of source datasets..28
Table D: Potential risk factors at the county level used in the BRT model for sandfly species and 2-stage XGBoost model for VL..29
Table E: Clustering analysis of model predictors at the county level based on pairwise Pearson correlation coefficients..30
Table F: Cross-tabulation of observed and XGBoost-model-predicted annual incidence levels of VL in 2016 31
Table G: BRT-model-estimated mean (standard deviation) relative contributions of top factors (RC ≥5%) to the spatial distribution of six most prevalent sandfly species in the *Phlebotomus* genus .32
Table H: BRT-model-estimated mean (standard deviation) relative contributions of top factors (RC ≥5%) to the spatial distribution of six most prevalent sandfly species in the *Sergentomyia* genus 33
Table I: Projections of the numbers, land areas, and population sizes of counties affected by VL risk areas according to SSP126 34
Table J: Projections of the numbers, land areas, and population sizes of counties affected by VL risk areas according to SSP245 35
Supplementary References 2 36

Supplementary Materials and Methods

Text A: Data Collection and Management

Database of Sandflies and Sandfly-associated Pathogens

We included only studies that reported clearly recognizable sandfly species and sandfly-associated pathogens. For ambiguous data, we contacted the authors to obtain the original data or remove them from our database if clarification was not possible.

We extracted county information or exact coordinates from each geographic location where sandflies or sandfly-associated pathogens were reported.

For our analyses, each record was defined at the county level, indicated as one specific sandfly species or specific SAP that was reported at least once in the same county (e.g., surveys in different townships within the same county). If presence of sandfly species was reported at a larger scale than the county level, e.g., at the provincial or municipal level, the data were only used for descriptive analysis, while not used for modeling. By applying the same method as above, we also collected data on sandfly-associated pathogens in animals. As a supplementation, we also extracted information on the sandfly-associated pathogens that had been uploaded to the GenBank database using the same criteria and method.

Climate data

We obtained historical and future climate data from the WorldClim website as GeoTiff files with a spatial resolution of 2.5' (http://www.worldclim.org). For the historical data, we generated bioclimatic variables as predictors for our models using the R package "dismo", based on the monthly maximum temperature, monthly minimum temperature, and precipitation data from 1980–2018. These bioclimatic variables have been widely used in ecological studies [1], which can better capture seasonal trends that are related to the physiological constraints of different sandfly species, as compared to traditional meteorological variables.

Due to the high degree of correlation among bioclimatic variables, we conducted a cluster analysis using the R package "NbClust" to analyze pairwise correlations. Specifically, we formed a binary distance matrix in which the distance between any pair of bioclimatic variables was designated as 0 if the absolute value of their correlation coefficient exceeded 0.8 and 1 otherwise. The optimal number of clusters was determined using the Krzanowski and Lai index [2]. Based on the average values of bioclimatic variables from 1980–2018, this analysis identified eight distinct clusters (Table E in S1 Appendix). Only one variable from each cluster was used for model-fitting.

We obtained mean values of bioclimatic variables for the periods 2021–2040, 2041–2060, and 2061–2080 from the WorldClim website, respectively. We referenced the performance of General Circulation Models (GCMs) in simulating the regional climatic response to rising Greenhouse Gas (GHG) concentration in China and selected three models: CNRM-CM6-1 (Centre National de Recherches Météorologiques (CNRM) for CIMP6), GISS-E2-1-G (Goddard Institute for Space Studies) and MRI-ESM2-0 (Meteorological Research Institute Earth System Model) [3]. Under each scenario, different GCMs generate varying predictions of bioclimatic variables within the region [4]. To balance this variability, we calculated the final value of bioclimatic variables as the mean of the predicted values generated by these three GCMs.

Terrain data

Terrain data were obtained from the Resource and Environment Science and Data Center (RESDC) (http://www.resdc.cn), with a spatial resolution of 250m. To accurately represent topography at the county scale, we additionally calculated the average and standard deviation of elevation, which were used as predictors in our modeling analysis (Table B in S1 Appendix).

Socioeconomic data

Gridded datasets about population projections under Shared Socioeconomic Pathways were obtained from the Science Data Bank, featuring a spatial resolution of 0.5° and a temporal range from 2010 to 2100. Using rural and total population data, we calculated population density, rural population density, and rural population proportion as predictors in our modeling analysis (Table B in S1 Appendix). All these variable measurements were calculated by matching the study time for modeling. The future data for variables at three-time points (2030, 2050, 2070) were used to predict the future changes. All data were processed into raster form using the R package "terra" and specific values for each variable were extracted at the county level.

Case data

The data were collected without any age or patient-based restrictions. To mitigate the influence of imported cases from within and outside China, we excluded case data from non-endemic provinces. Endemic provinces were defined as those with a history of confirmed local cases of VL in the past 10 years through epidemiological case investigations. Non-endemic provinces were defined as those without records of local distribution of the sandfly vectors for VL transmission or without confirmed

local cases of VL in the past 10 years through epidemiological investigations [5, 6].

Map data

All the base layers of the maps are sourced from the RESDC (https://www.resdc.cn/DOI/DOI.aspx?DOIID=120) [7].

Text B: Ecological modeling and clustering analysis of sandflies

Ecological Modeling of sandflies

To explore the relationship between the environmental suitability of sandflies and county-level variable values, we used the boosted regression tree (BRT) methodology. BRT is a predictive learning algorithm that combines traditional regression tree or classification tree with gradient boosting, which effectively models complex response functions while avoiding overfitting. This method is widely used for vector distribution mapping [8, 9].

$$y_i = F(x_i) = \sum_{m=0}^{M} \beta_m h(x_i; \alpha_m)$$

where y_i is the outcome and x_i is the vector of predictors for individual observation i, $i = 1,2,\cdots,n$. M is the number of trees, $h(x_i; \alpha_m)$ returns the terminal node of a tree defined by parameters α_m (representing both selected predictors and splitting values) for an input x_i, and β_m is the expansion coefficient. Starting from some initial estimation of $F(x_i)$, $F_0(x_i)$, the tree structures and coefficients are determined sequentially by the following optimization for $m = 1,2,\cdots,M$,

$$(\beta_m, \alpha_m) = \underset{\beta,\alpha}{\operatorname{argmin}} \sum_{i=1}^{N} L[y_i, F_{m-1}(x_i) + \beta h(x_i; \alpha)]$$

where $F_m(\boldsymbol{x_i}) = F_{m-1}(\boldsymbol{x_i}) + \beta_m h(\boldsymbol{x_i}; \boldsymbol{\alpha_m})$, and $L(y_i, F)$ is the loss function.

Similar to other ecological models, the BRT model requires defining presence and absence data as response variables. To reduce the sampling bias for the surveyed counties, we established a logistic regression model to predict the sampling probabilities of all the counties with the 13 selected variables used as predictors. The reciprocals of the predicted sampling probabilities of all the surveyed counties were rescaled to have a mean value of 1 and the rescaled values were subsequently used as weights in the BRT models [10-12]. The response variable was assigned “1” for the counties that had been surveyed and 0 for the unsurveyed. A backward selection procedure was employed to choose the variables at a significance level of 0.05.

Model parameters were determined based on the satisfactory performance observed in our previous study, with a tree complexity of 5, a learning rate of 0.005, and a bagging rate of 75% for the primary analysis [13-15]. We performed 10-fold cross-validation using the “gbm.step” function available in the R package “dismo” to ascertain the optimal number of trees. To quantify model uncertainty and enhance model robustness, we constructed an ensemble model comprising 100 individual BRT models.

To provide a more robust and parsimonious estimation of model parameters, a two-stage bootstrapping procedure was employed. Within each stage, the following split-and-fit step was iteratively executed for a predetermined number of repetitions. A training set comprising 75% of data points was randomly selected via bootstrapping without replacement, and the remaining 25% served as a test set. The BRT model was constructed using the training set and subsequently applied to the test set for validation when necessary. The output generated by the BRT model includes both predicted environmental suitability and relative contribution (RC) (or influence) of each variable. The RC is calculated based on the frequency with which a variable is selected for splitting and the degree to which each splitting improves the objective function averaging over all trees. The RCs of all variables are normalized such that their summation equals 1 [16]. At the first stage, the split-fitting step was iteratively executed ten times to screen for significant variables. At this stage, the trained model was not validated using the test set. All variables with RC <2% from the bootstrap training set were excluded from the next stage. At the second stage, the split-fitting step was repeated 100 times using the remaining variables. Since no variable selection was performed at this stage, all 100 models contained the same variables but produced different contribution estimates. The final RCs of the variables are the average that were calculated from all 100 BRT models. The receiver operating characteristic (ROC) curves and areas under curve (AUC) based on the test sets were also averaged to represent the final predictive performance. The standard deviations and 95% percentiles of the RCs and AUCs across all 100 models were employed to quantify the uncertainty in the estimation. Considering the potential false negative and false positive counties within observed data, partial area AUCs with a tolerance level of 0.2 for omission error were also calculated. For partial area AUCs, the horizontal axis represents the total rate of positives rather than false positives. The ratio of partial AUC to the area under the random selection line (diagonal line) was presented as suggested by Peterson et al [17]. We also conducted a sensitivity analysis using a learning rate of 0.01 for selected sandfly species. However, our findings indicated no significant deviation in the contribution estimates. Owing to the substantial

volume of data (comprising 14 predictors) and the number of models runs (encompassing 12 sandflies and 100 iterations), we cannot afford a complete cross-validation optimization for all model. BRT modeling was conducted using the R packages "dismo" and "gbm", and predictive performance was assessed using the R packages "ROCR" and "pROC" in the R v4.1.2 (R Core Team, 2021).

Clustering of sandflies with similar ecological niches and their spatial distribution.

We generated features for clustering by the following steps. First, we eliminated predictors that were non-influential, i.e., not remained in final models, for all the 12 sandfly species. Second, we computed three properties for each remaining predictor and each sandfly species: i) the average relative contribution of the predictor in the final 100 BRT models, or zero if the predictor was not in the final models for that sandfly species; ii) the difference of the predictor between case counties (positive for that sandfly species) and all counties, measured by the quartile location (1–4) of the median value of the predictor among case counties relative to all counties; and iii) the linear correlation between the predictor and the model-predicted presence probabilities of the sandfly species among all counties (averaged over 100 models). Finally, we used these three indicators as features for clustering.

Text C: Ecological Modeling of VL

Ecological Modeling of VL

Among the machine learning methods used in practice, gradient boosted regression tree (GBRT) is one technique that shines in many applications. We utilized the implementation of GBRT available in the R package "xgboost" [18]. Besides the same variables as in sandfly species distribution model, we included four sandfly species used as variables to account for the influence of vectors on VL incidence (Table B in S1 Appendix). The screening process for all variables was conducted like in the sandfly BRT model. We matched variable values by year for each county to accurately capture characteristic information.

At the first stage, a logistic extreme gradient boosting (XGBoost) model was employed to fit the presence or absence of VL cases in each county. All counties where human cases were reported during surveillance from 2014–2018 were designated as "presence", and the remaining counties were designated as "absence". The modeling process was first repeated ten times with all predictors included, then predictors with RCs <2% were excluded. Subsequently, the modeling was repeated 100 times, and the final results (estimated RCs and response curves) were averaged over 100 models. Any county with a predicted VL suitability value above the threshold was considered at risk. This stage accounts for the excessive amount of zero case numbers in the majority of the nation.

At the second stage, counties with a non-zero average annual incidence of reported human cases from 2014 to 2018 were fitted with the XGBoost model. This model assumed that VL incidence adheres to a gamma distribution. The gamma distribution was selected due to it best fits the observed non-zero average VL annual incidence. Like the first-stage predictor screening process, we first repeated the modeling five times with all predictors included, then excluded predictors with RCs <2%. The model fitting process was repeated five times, each time with one of the years 2014–2018 as the testing data and the remaining years as the training data. The results (estimated relative contributions and response curves) are averaged over the five models. Two-stage model parameters were set to a learning rate of 0.1, a maximum tree depth of 5, and a bag fraction of 0.8 for the run. The optimal number of trees (referred to as "nrounds" in xgboost) was determined by 5-fold cross-validation. The XGBoost models were developed using the "xgboost" package in R v4.1.2 (R Core Team. 2021).

Supplement References 1

1 O'Donnell MS, Ignizio DA. Bioclimatic predictors for supporting ecological applications in the conterminous United States. US Geol Surv Data Ser. 2012;691:4–9.
2 Miao D, Liu MJ, Wang YX, Ren X, Lu QB, Zhao GP, et al. Epidemiology and Ecology of Severe Fever With Thrombocytopenia Syndrome in China, 2010–2018. Clin Infect Dis. 2021;73(11):e3851-e8.
3 Yang X, Gao Z, Wang L, Xiao L, Dong N, Wu H, et al. Projecting the potential distribution of ticks in China under climate and land use change. Int J Parasitol. 2021;51(9):749-59.
4 Kraemer MUG, Reiner RC, Jr., Brady OJ, Messina JP, Gilbert M, Pigott DM, et al. Past and future spread of the arbovirus vectors Aedes aegypti and Aedes albopictus. Nat Microbiol. 2019;4(5):854-63.
5 Zheng-bin Z, Yuan-yuan L, Yi Z, Shi-zhu L. Prevalence of visceral leishmaniasis in China during 2015-2018. Chin J Parasitol Parasit Dis. 2020;38(3):339.
6 Han S, Wu W, Xue C, Ding W, Hou Y, Feng Y, et al. Endemic status of visceral leishmaniasis in China from 2004 to 2016. Chin J Parasitol Parasit Dis. 2019;37(2):189-95.
7 XL Xu. Multi-year county-level administrative boundary data of China. Resource and Environment Science Data Registration and Publishing System. (http://www.resdc.cn/DOI), 2023.
8 Stevens KB, Pfeiffer DU. Spatial modelling of disease using data- and knowledge-driven approaches. Spatial Spatio-temporal Epidemiol. 2011;2(3):125-33.
9 Krzywinski M, Altman N. Classification and regression trees. Nat Methods. 2017;14(8):757-8.
10 Pandit PS, Doyle MM, Smart KM, Young CCW, Drape GW, Johnson CK. Predicting wildlife reservoirs and global vulnerability to zoonotic Flaviviruses. Nat Commun. 2018;9(1):5425.
11 Albery GF, Eskew EA, Ross N, Olival KJ. Predicting the global mammalian viral sharing network using phylogeography. Nat Commun. 2020;11(1):2260.
12 Little RJA, Rubin DB. Statistical Analysis with Missing Data. John Wiley & Sons: Hoboken; 2002.
13 Yao H, Wang Y, Mi X, Sun Y, Liu K, Li X, et al. The scrub typhus in mainland China: spatiotemporal expansion and risk prediction underpinned by complex factors. Emerging Microbes Infect. 2019;8(1):909-19.
14 Zhao GP, Wang YX, Fan ZW, Ji Y, Liu MJ, Zhang WH, et al. Mapping ticks and tick-borne pathogens in China. Nat Commun. 2021;12(1):1075.
15 Wang T, Fan ZW, Ji Y, Chen JJ, Zhao GP, Zhang WH, et al. Mapping the Distributions of Mosquitoes and Mosquito-Borne Arboviruses in China. Viruses. 2022;14(4).
16 Elith J, Leathwick JR, Hastie T. A working guide to boosted regression trees. J Anim Ecol. 2008;77(4):802-13.
17 Peterson AT, Papeş M, Soberón J. Rethinking receiver operating characteristic analysis applications in ecological niche modeling. Ecol Model. 2008;213(1):63-72.
18 Chen T, Guestrin C, editors. XGBoost: A Scalable Tree Boosting System. Proceedings of the 22nd ACM SIGKDD International Conference on Knowledge Discovery and Data Mining; 2016.

Supplementary Figures

Fig A: The spatial distribution of the 673 counties with at least one record of sandflies (yellow) from 1940 to 2022, China. Base layers of the maps were downloaded from Resource and Environment Science and Data Center (https://www.resdc.cn/DOI/DOI.aspx?DOIID=120).

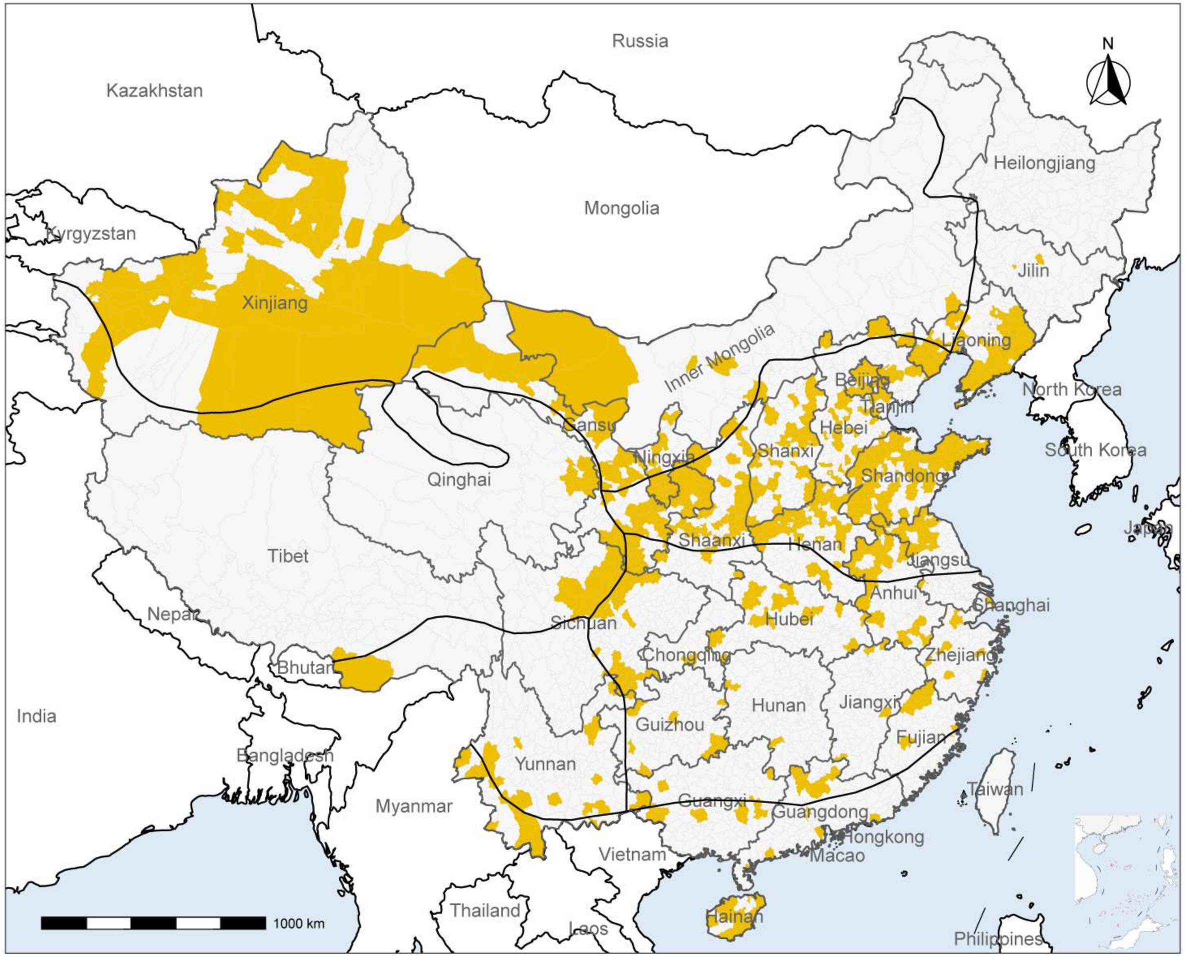

Fig B: The number of records related to sandflies in different provinces in different periods (with the number of publications in parentheses) and a comparison of the total number of records for four VL vector sandflies in each province.

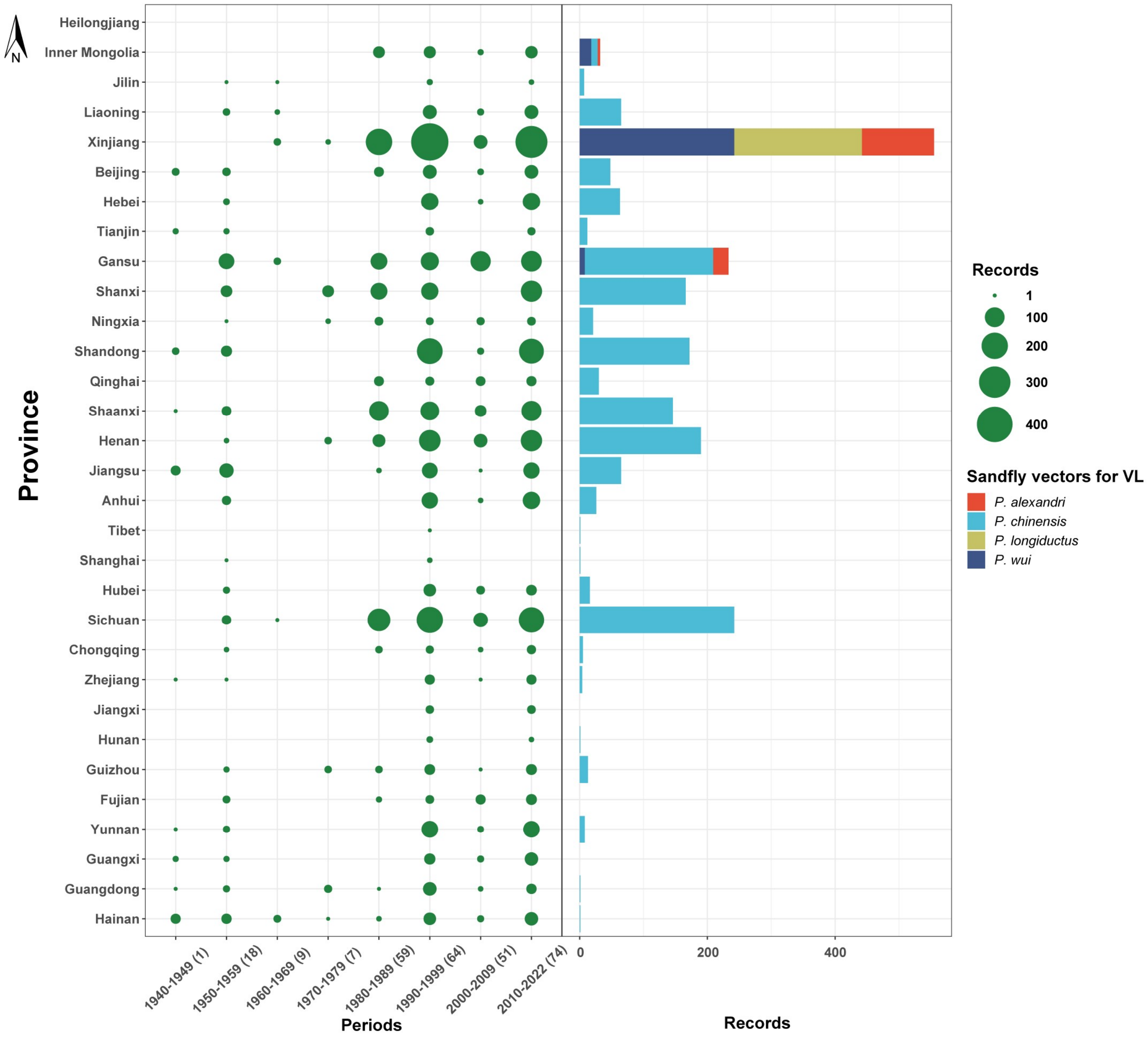

Fig C: The spatial distribution of the sandfly genus Phlebotomus recorded at the county level from 1940 to 2022 in China. Base layers of the maps were downloaded from Resource and Environment Science and Data Center (https://www.resdc.cn/DOI/DOI.aspx?DOIID=120).

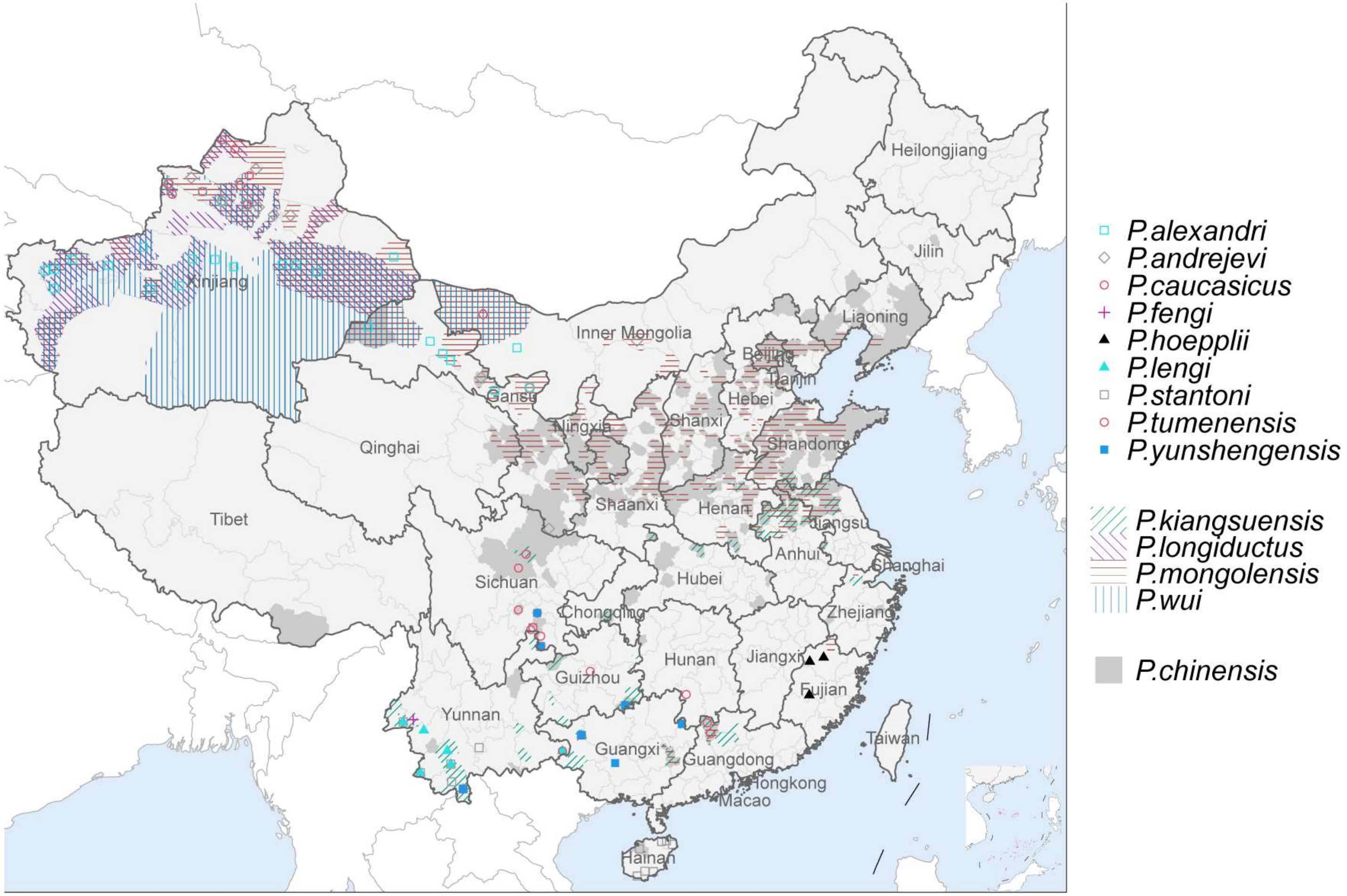

Fig D: The spatial distribution of the sandfly genus *Sergentomyia* recorded at the county level from 1940 to 2022 in China. Base layers of the maps were downloaded from Resource and Environment Science and Data Center (https://www.resdc.cn/DOI/DOI.aspx?DOIID=120).

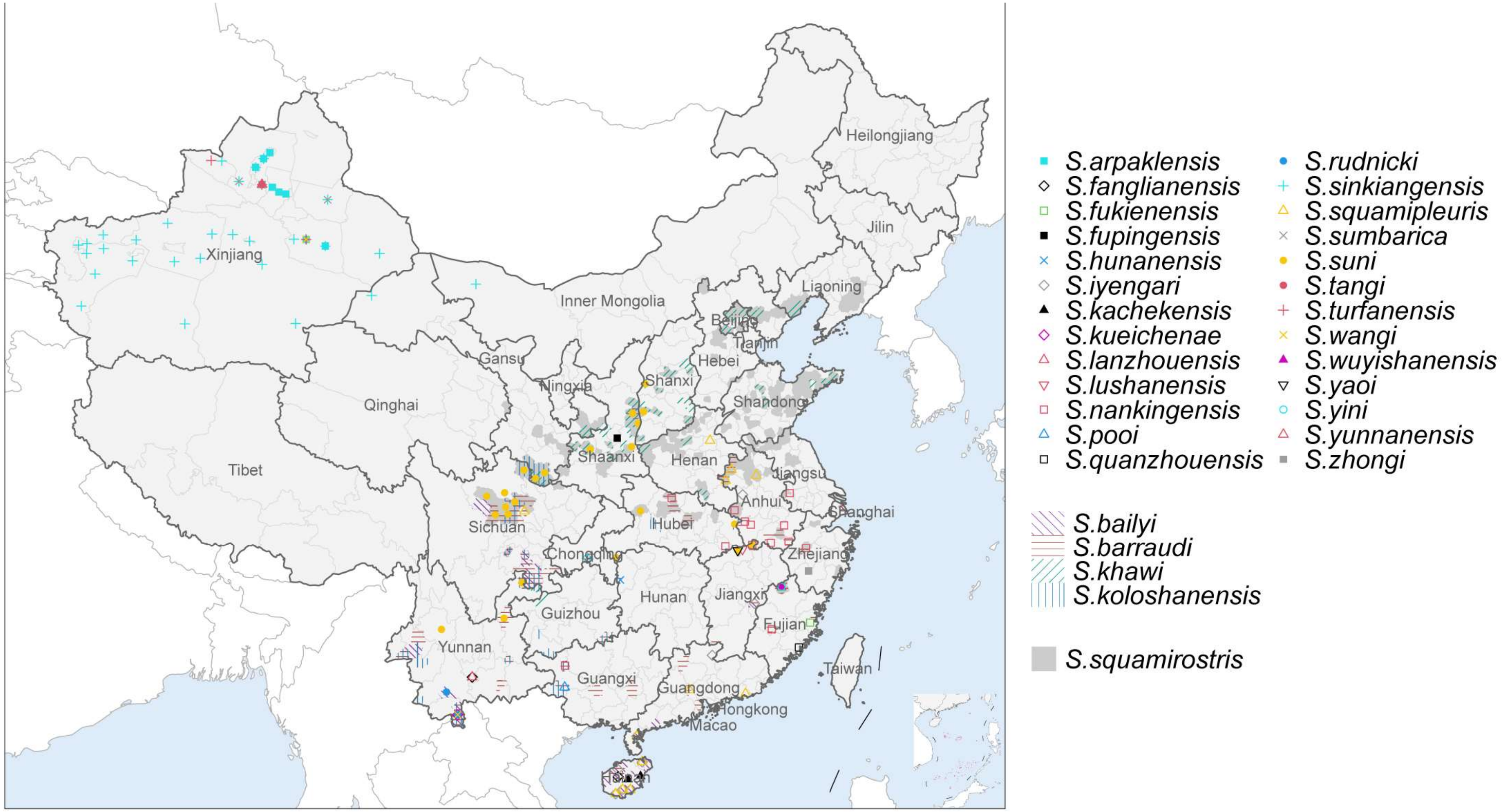

Fig E: The spatial distribution of the sandfly genus *Chinius* and *Idiophlebotomus* recorded at the county level from 1940 to 2022 in China. Base layers of the maps were downloaded from Resource and Environment Science and Data Center (https://www.resdc.cn/DOI/DOI.aspx?DOIID=120).

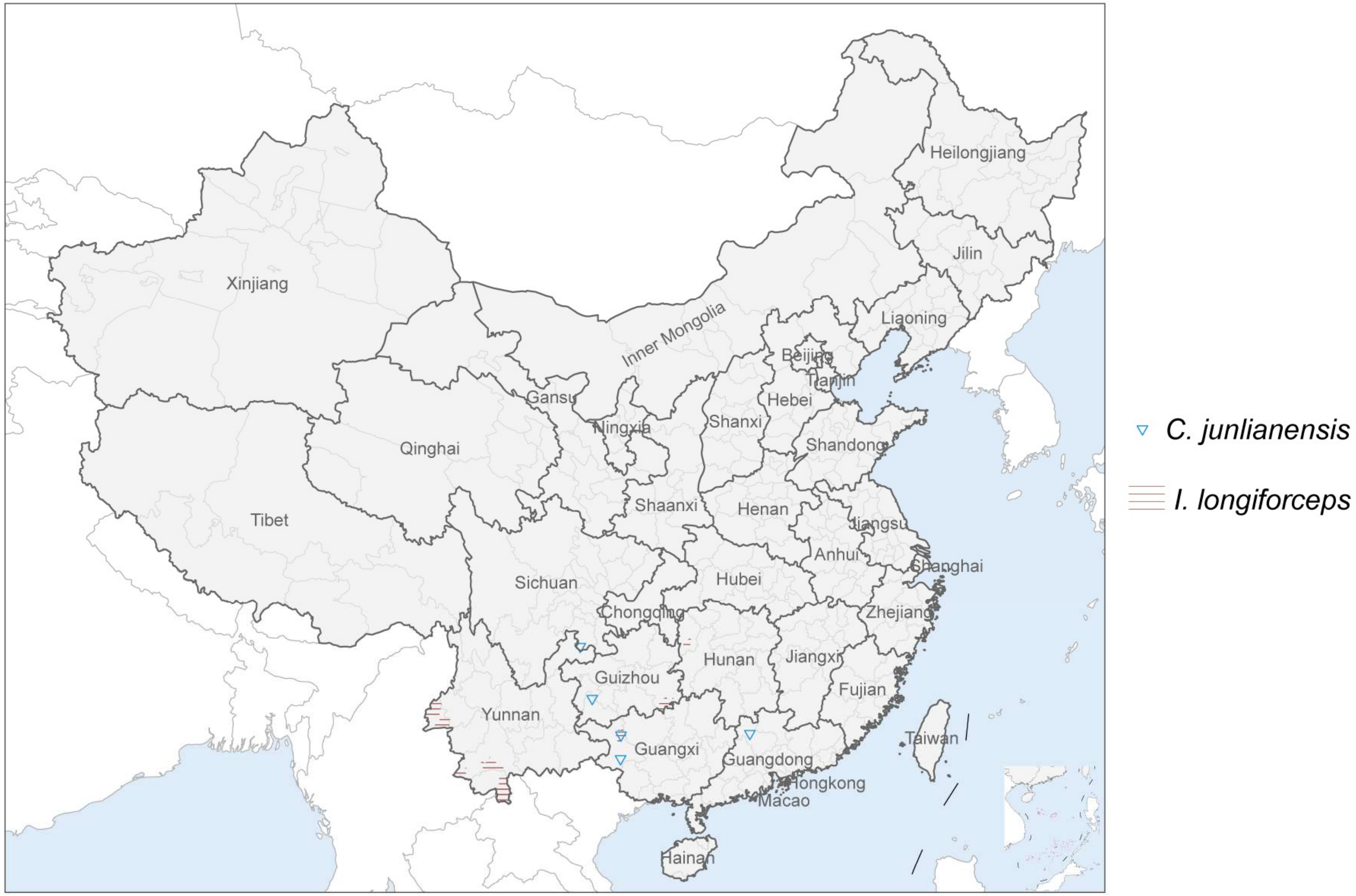

Fig F: Sandfly species richness (circles) at the prefecture level in seven biogeographic zones in the mainland of China from 1940 to 2022. Base layers of the maps were downloaded from Resource and Environment Science and Data Center (https://www.resdc.cn/DOI/DOI.aspx?DOIID=120).

I=Northeast district, II=North China district, III=Inner Mongolia-Xinjiang district, IV= Qinghai-Tibet district, V=Southwest China district, VI=Central China district and VII=South China district.

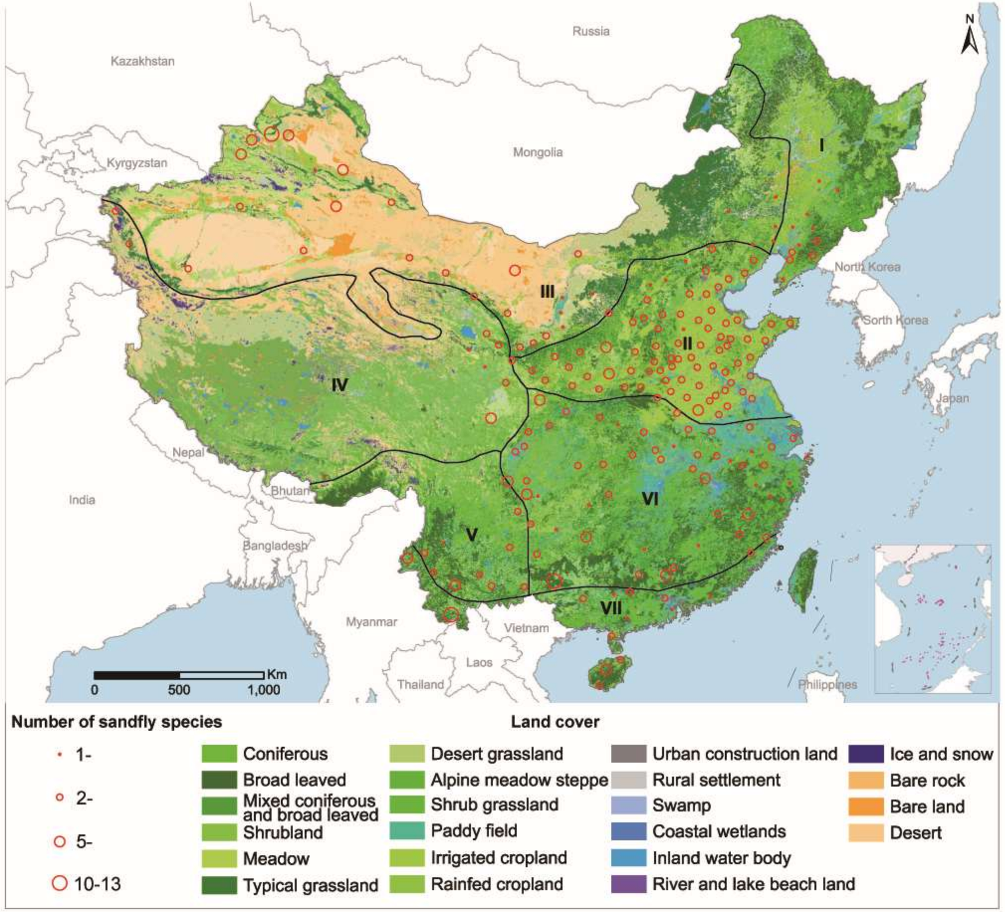

Fig G: The predicted county-level distributions of the six most prevalent sandfly species in the *Phlebotomus* genus, averaged over the ensemble of BRT models. Base layers of the maps were downloaded from Resource and Environment Science and Data Center (https://www.resdc.cn/DOI/DOI.aspx?DOIID=120).
(A) P. chinensis, (B) P. mongolensis, (C) P. kiangsuensis, (D) P. wui, (E) P. longiductus, (F) P. alexandri.

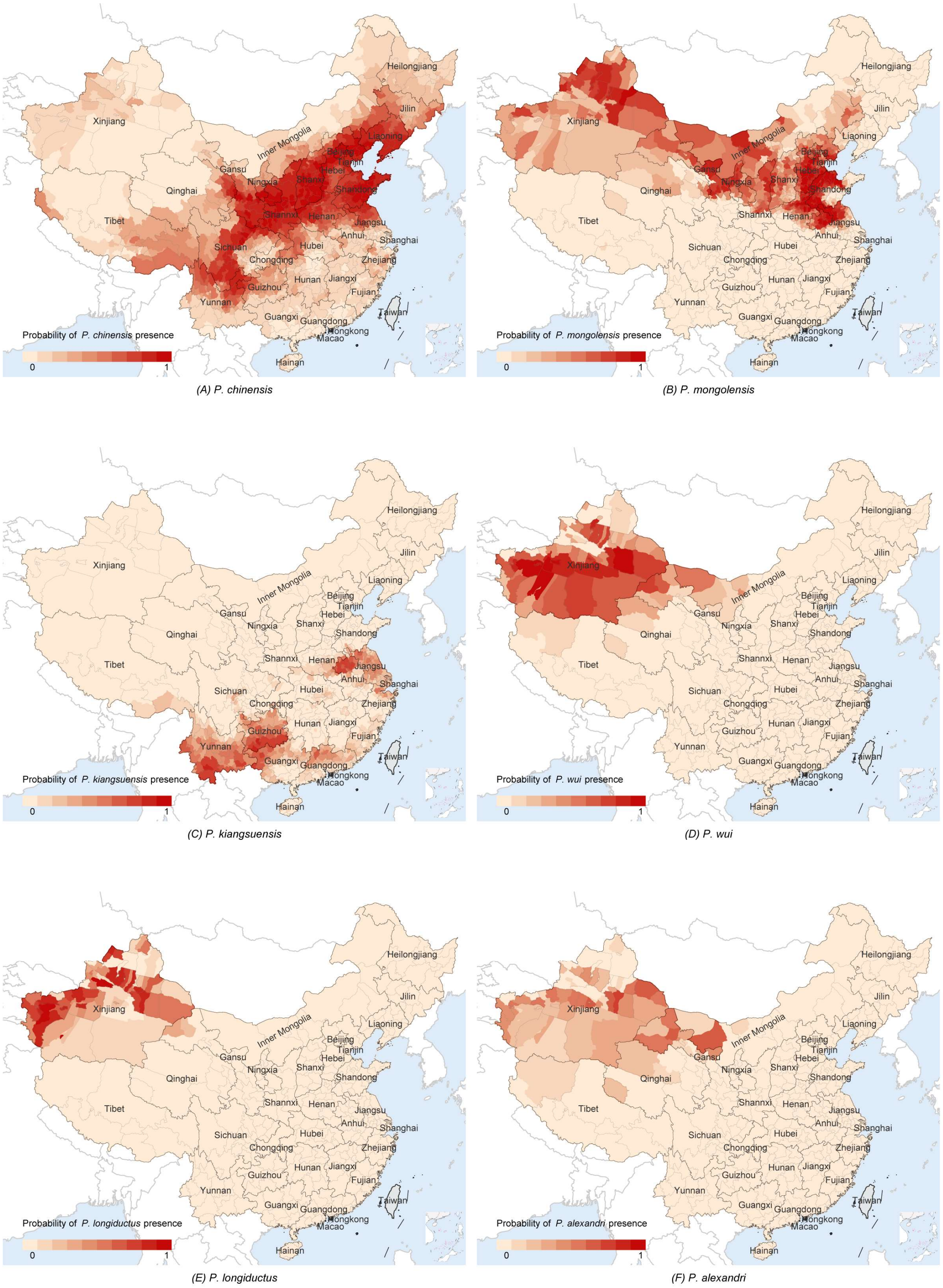

(A) P. chinensis
(B) P. mongolensis
(C) P. kiangsuensis
(D) P. wui
(E) P. longiductus
(F) P. alexandri

Fig H: The predicted county-level distributions of the six most prevalent sandfly species in the *Sergentomyia* genus, averaged over the ensemble of BRT models. Base layers of the maps were downloaded from Resource and Environment Science and Data Center (https://www.resdc.cn/DOI/DOI.aspx?DOIID=120).

(A) S. squamirostris, (B) S. barraudi, (C) S. khawi, (D) S. koloshanensis, (E) S. sinkiangensis, (F) S. sinkiangensis.

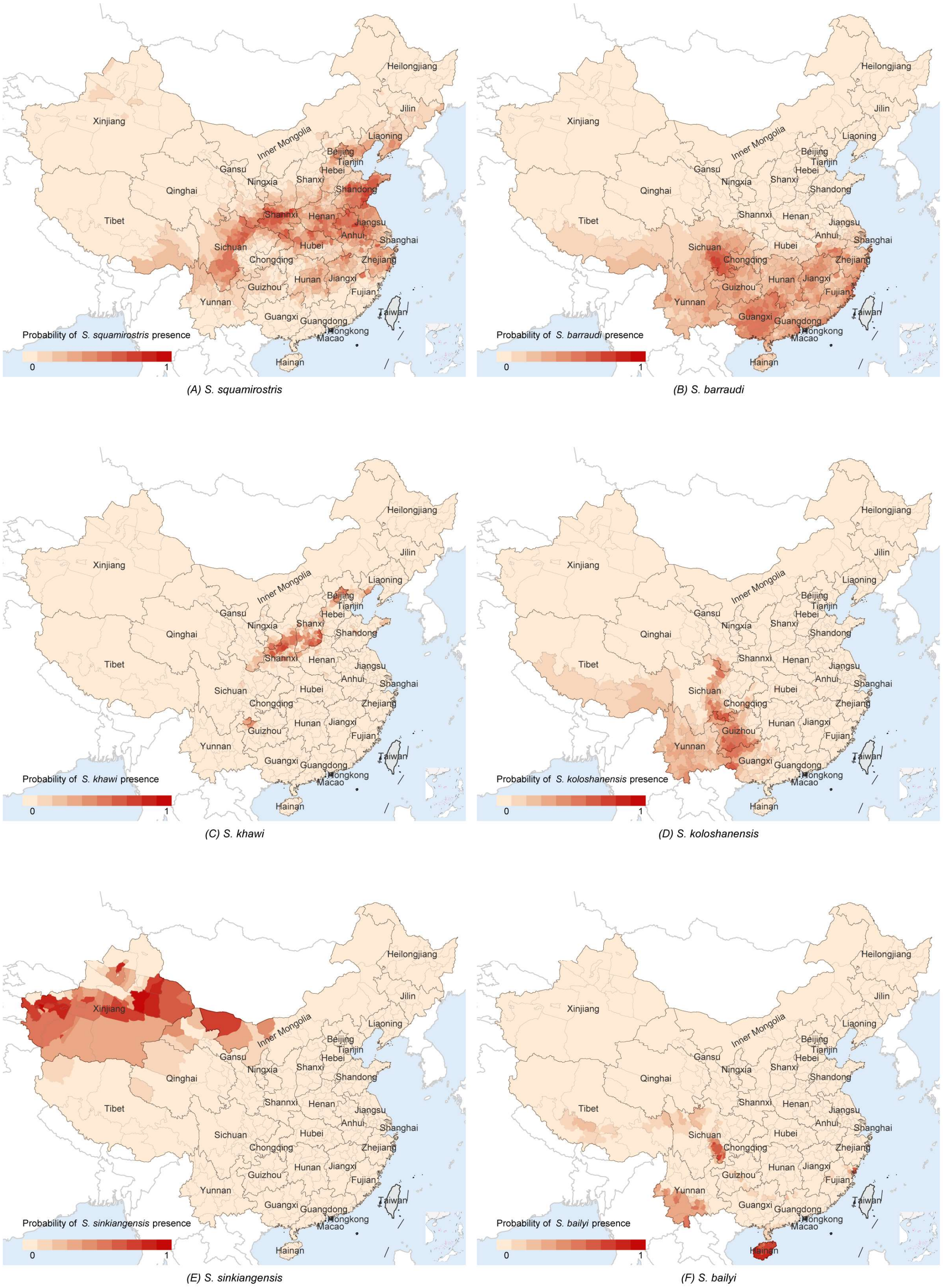

Fig I: The mean curves (red) and 95% percentiles (gray) for the effects of major predictors (RC ≥5%) on the probability of occurrence of 12 main sandfly species based on the ensemble of BRT models.

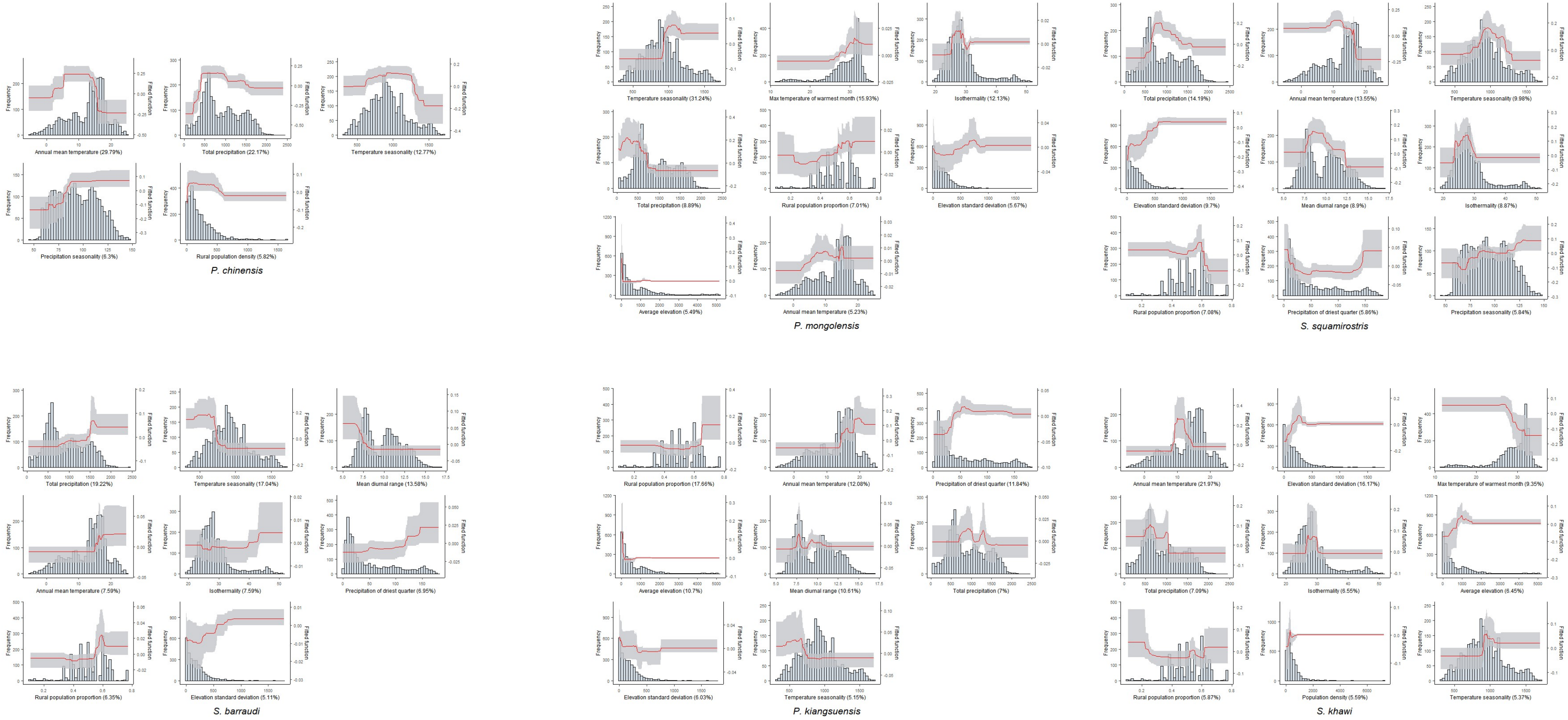

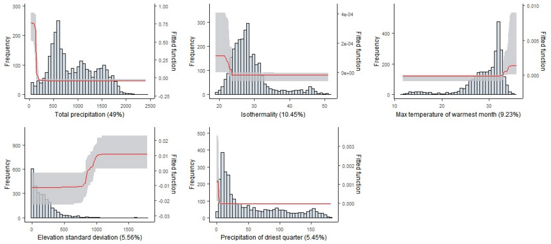

S. sinkiangensis

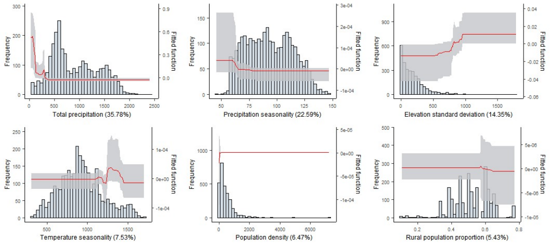

P. longiductus

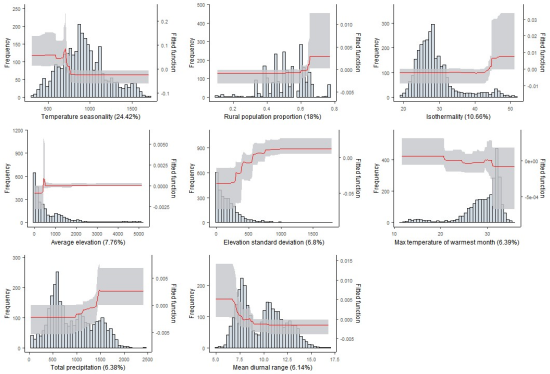

S. koloshanensis

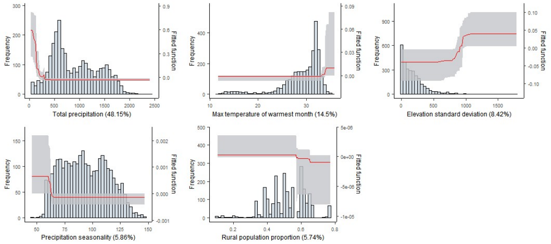

P. wui

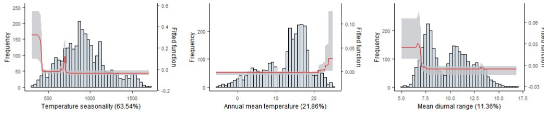

S. bailyi

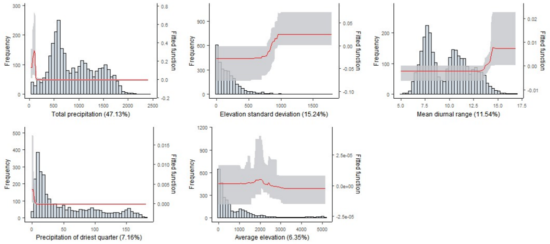

P. alexandri

Fig J: XGBoost-model-predicted AVL and DT-ZVL incidence in response to major predictors (RC ≥5%) when other predictors are fixed at mean values. The red curves and gray bands show the average and range, respectively, of predicted incidences from five XGBoost models, each with one of the years 2014–2018 as the testing set and the remaining years as the training set.

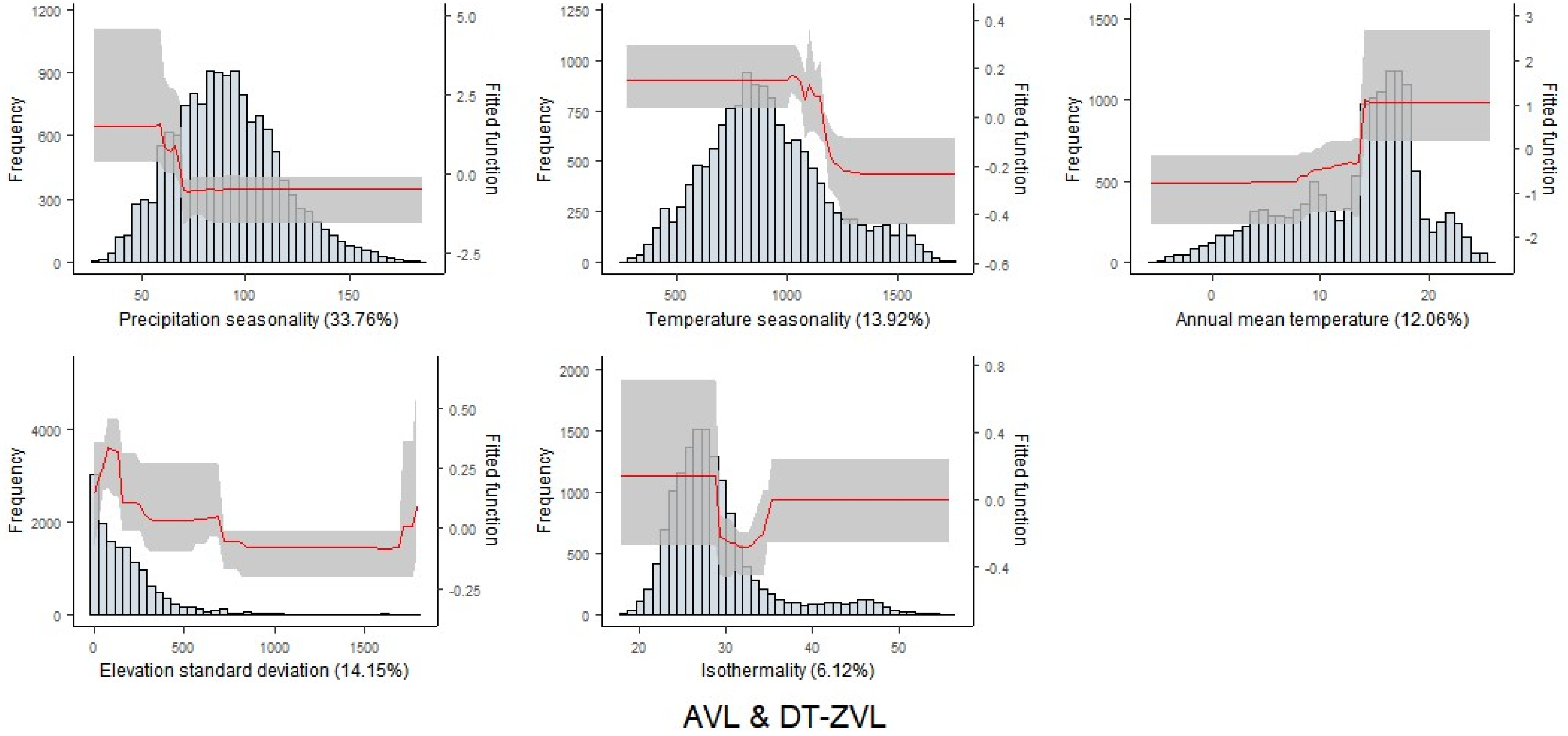

Fig K: XGBoost-model-predicted MT-ZVL incidence in response to major predictors (RC ≥5%) when other predictors are fixed at mean values.
The red curves and gray bands show the average and range, respectively, of predicted incidences from five XGBoost models, each with one of the years 2014–2018 as the testing set and the remaining years as the training set.

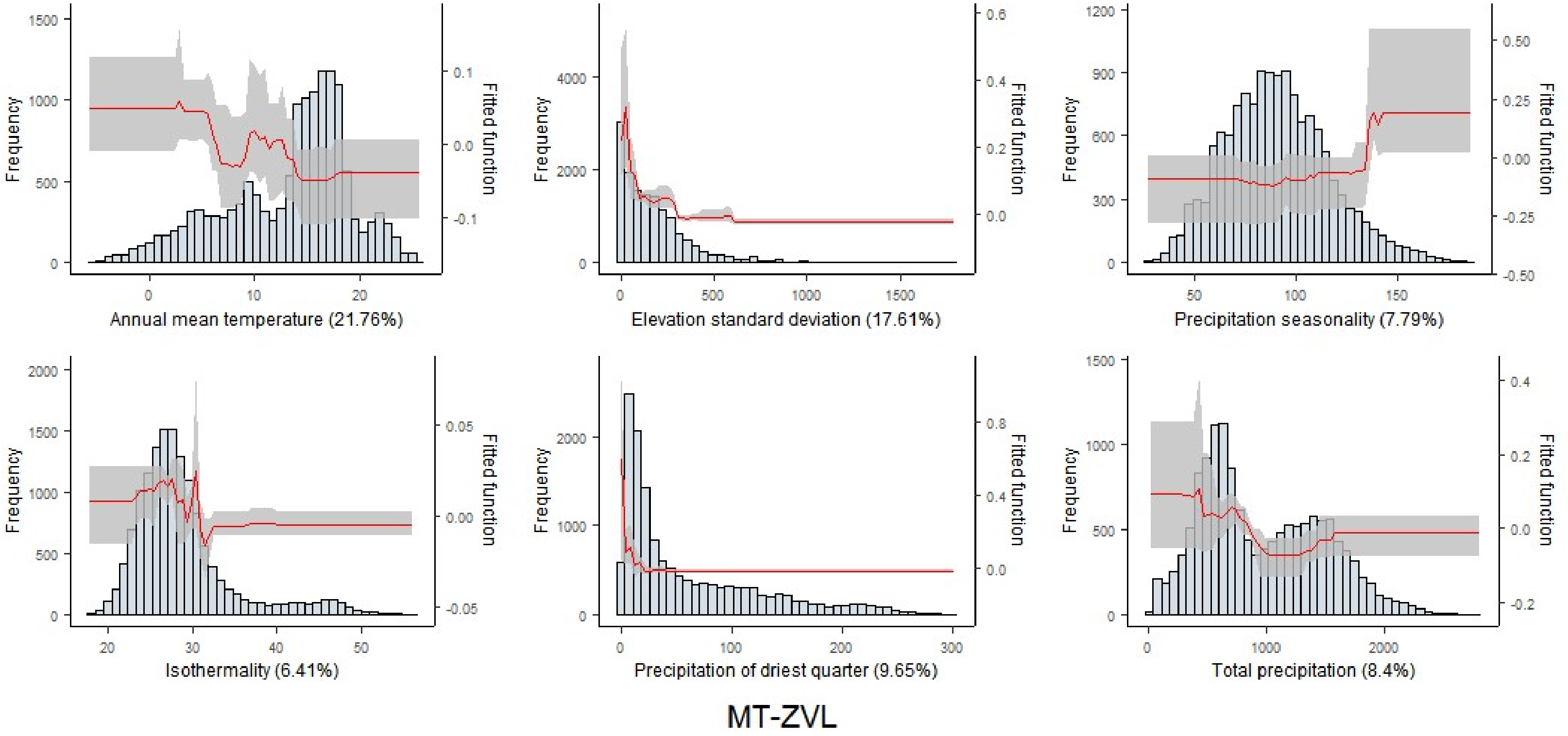

Fig L: Spatial distribution and changes in model-predicted incidence of VL in the mainland of China under SSP126. Base layers of the maps were downloaded from Resource and Environment Science and Data Center (https://www.resdc.cn/DOI/DOI.aspx?DOIID=120).
Panels a–c, Predicted annual incidence of VL in 2021–2040, 2041–2060, and 2061–2080, respectively. **Panels d–f,** Changes in predicted VL risk level from 2014–2018 to 2021–2040, from 2021–2040 to 2041–2060, and from 2041–2060 to 2061–2080, respectively.

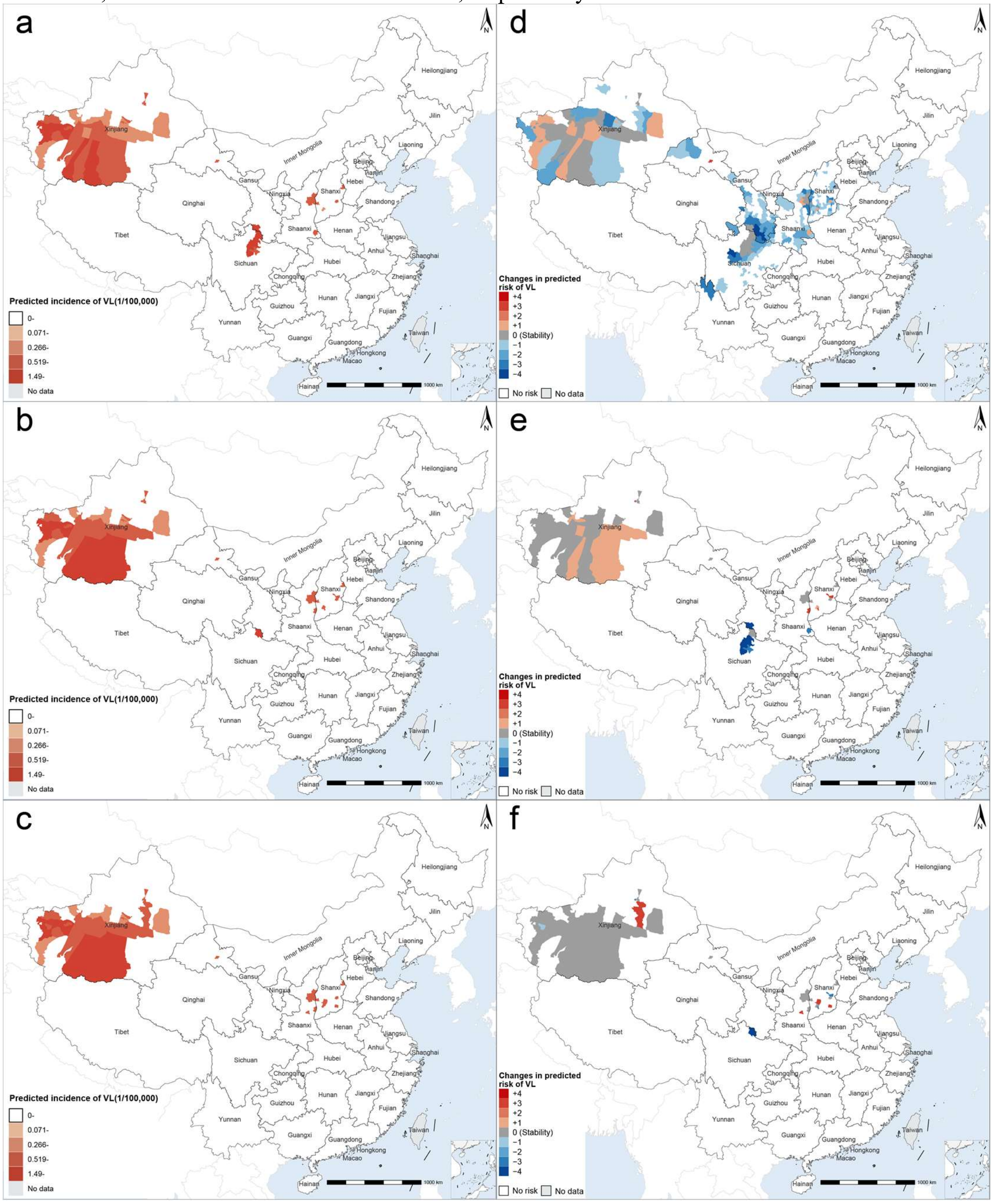

Fig M: Spatial distribution and changes in model-predicted incidence of VL in the mainland of China under SSP245. Base layers of the maps were downloaded from Resource and Environment Science and Data Center (https://www.resdc.cn/DOI/DOI.aspx?DOIID=120).
Panels a–c, Predicted annual incidence of VL in 2021–2040, 2041–2060, and 2061–2080, respectively.
Panels d–f, Changes in predicted VL risk level from 2014–2018 to 2021–2040, from 2021–2040 to 2041–2060, and from 2041–2060 to 2061–2080, respectively.

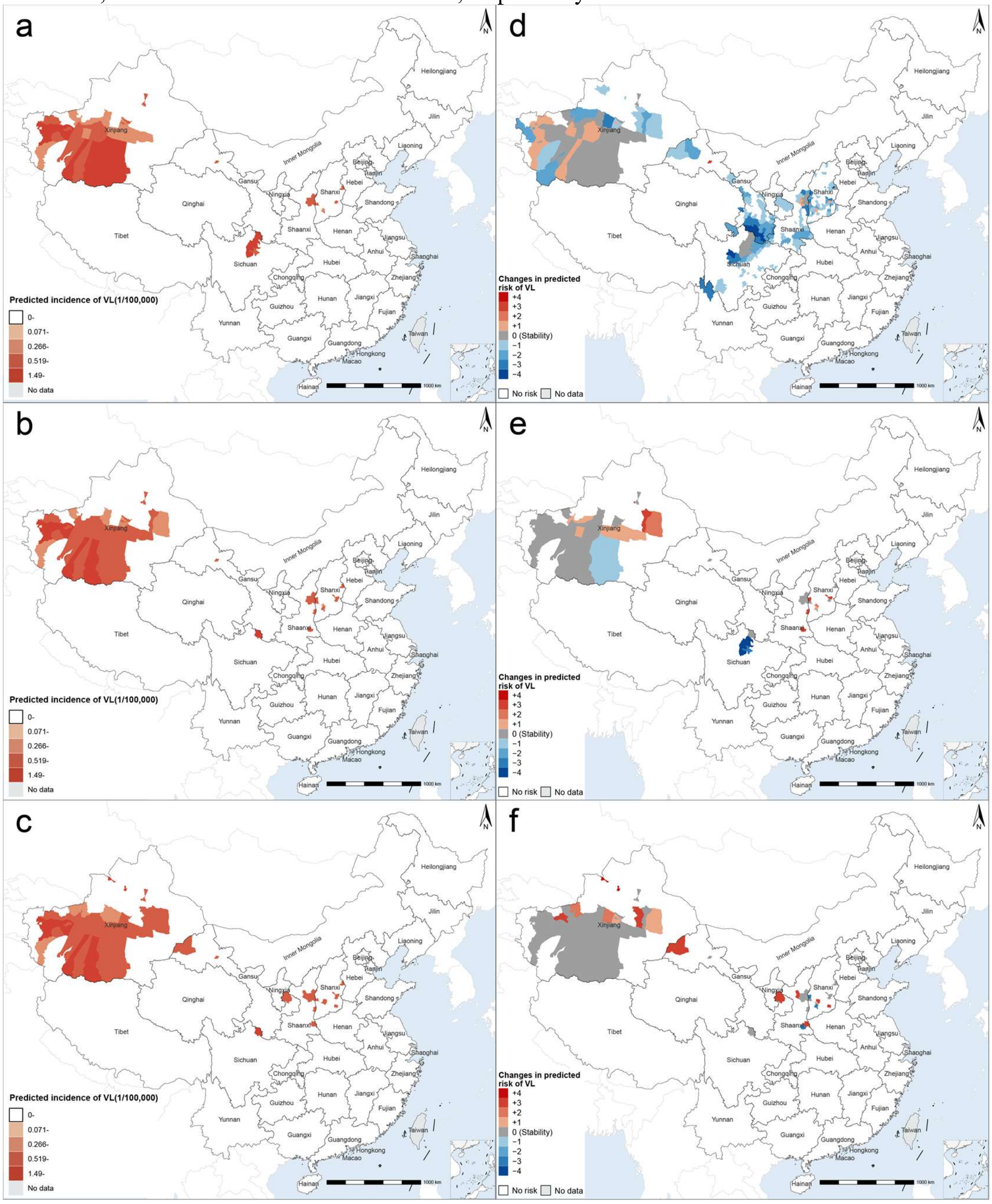

Fig N: Spatial distribution and changes in model-predicted environmental suitability of *P. wui* in future under three scenarios. Base layers of the maps were downloaded from Resource and Environment Science and Data Center (https://www.resdc.cn/DOI/DOI.aspx?DOIID=120).

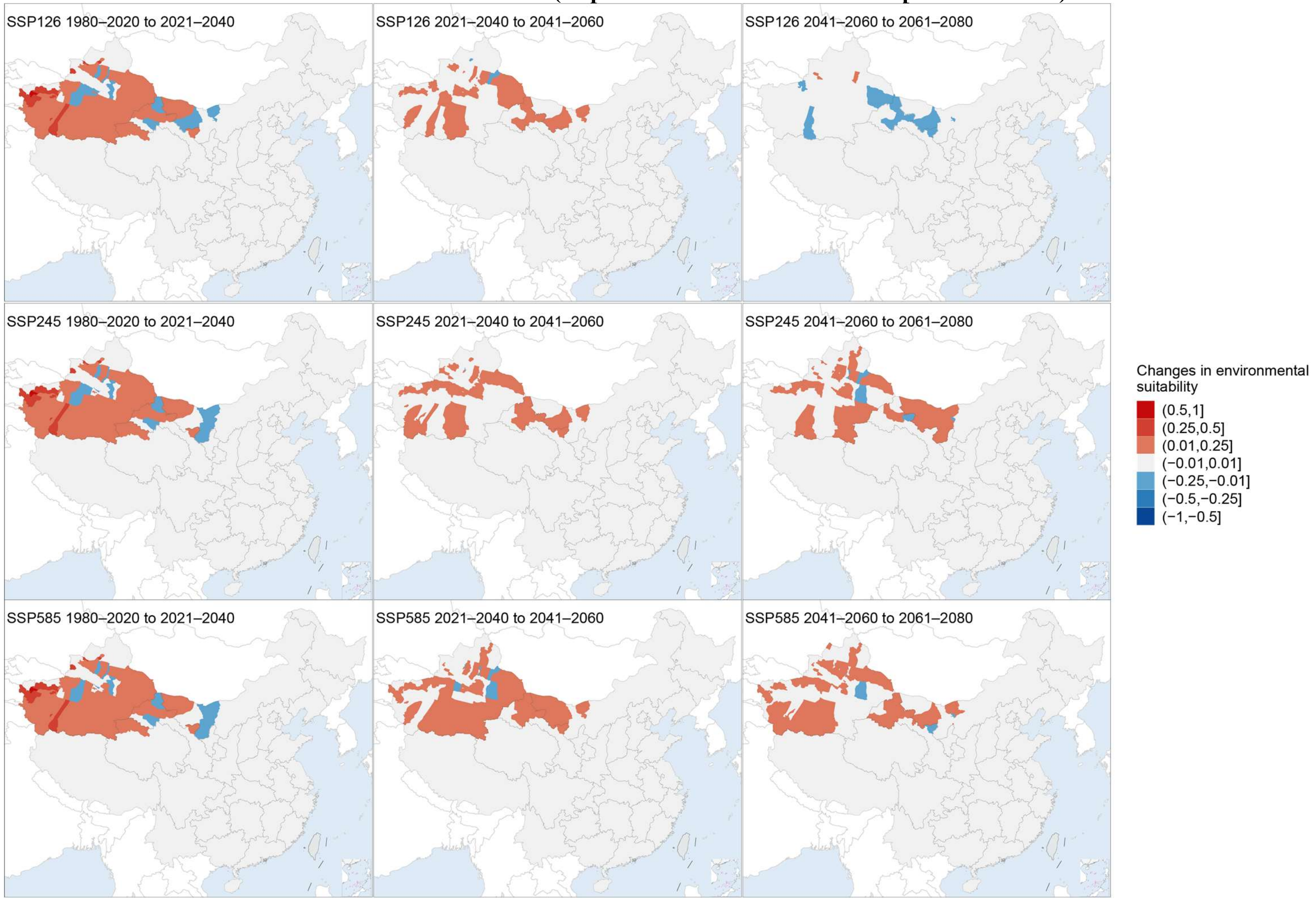

Fig O: Spatial distribution and changes in model-predicted environmental suitability of *P. chinensis* in future under three scenarios. Base layers of the maps were downloaded from Resource and Environment Science and Data Center (https://www.resdc.cn/DOI/DOI.aspx?DOIID=120).

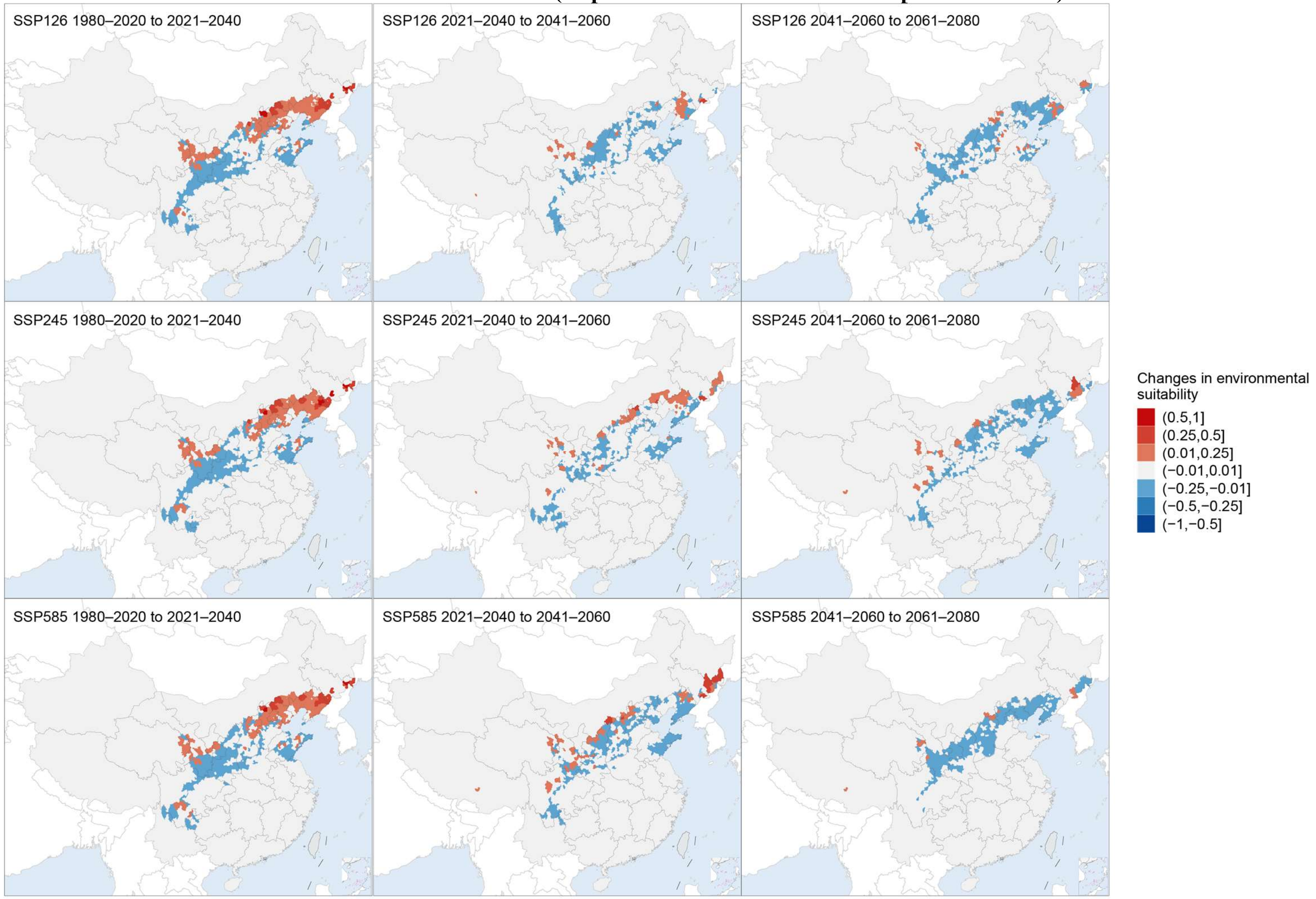

Fig P: Spatial distribution and changes in model-predicted environmental suitability of *P. longiductus* in future under three scenarios. Base layers of the maps were downloaded from Resource and Environment Science and Data Center (https://www.resdc.cn/DOI/DOI.aspx?DOIID=120).

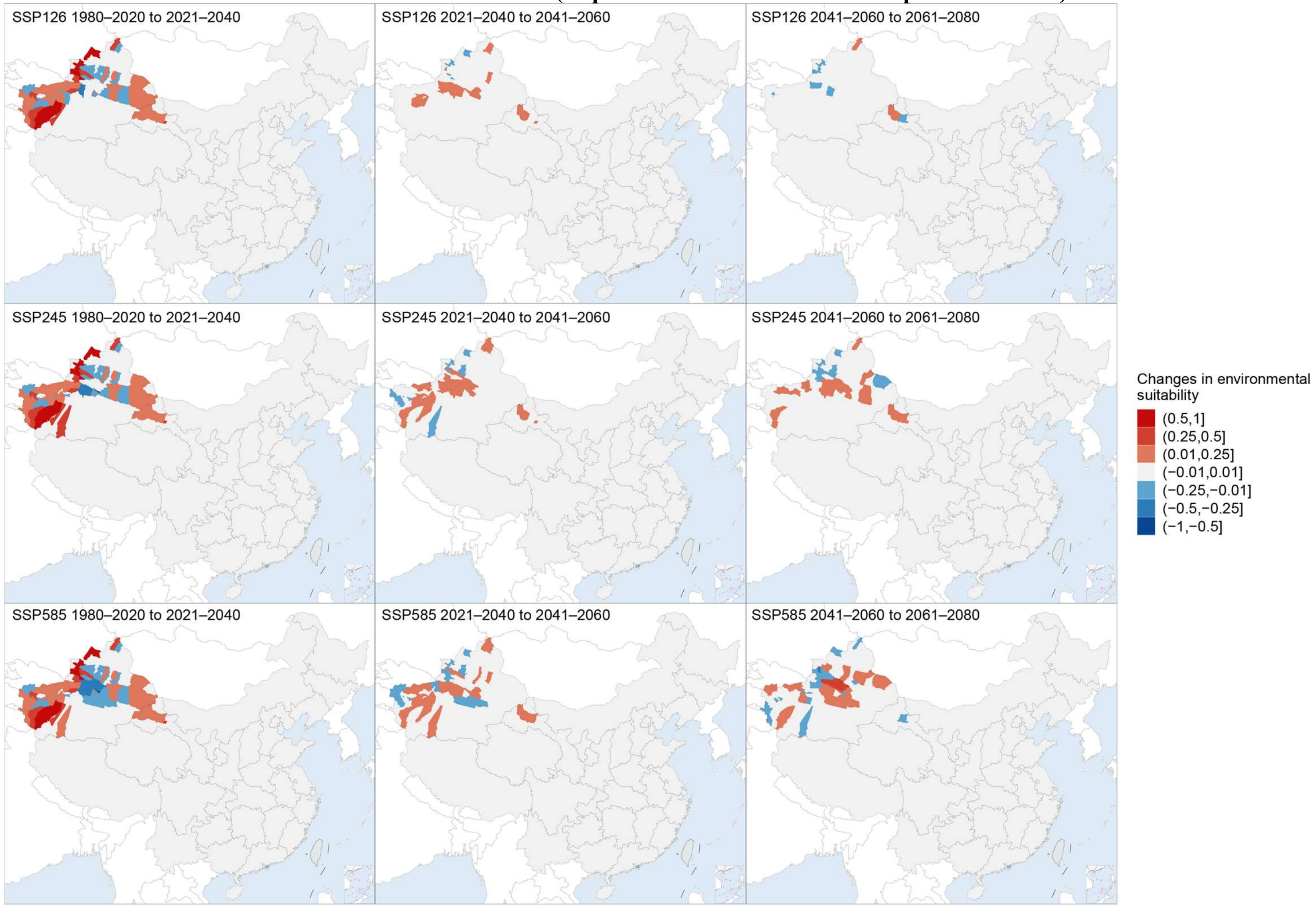

Fig Q: Spatial distribution and changes in model-predicted environmental suitability of *P. alexandri* in future under three scenarios. Base layers of the maps were downloaded from Resource and Environment Science and Data Center (https://www.resdc.cn/DOI/DOI.aspx?DOIID=120).

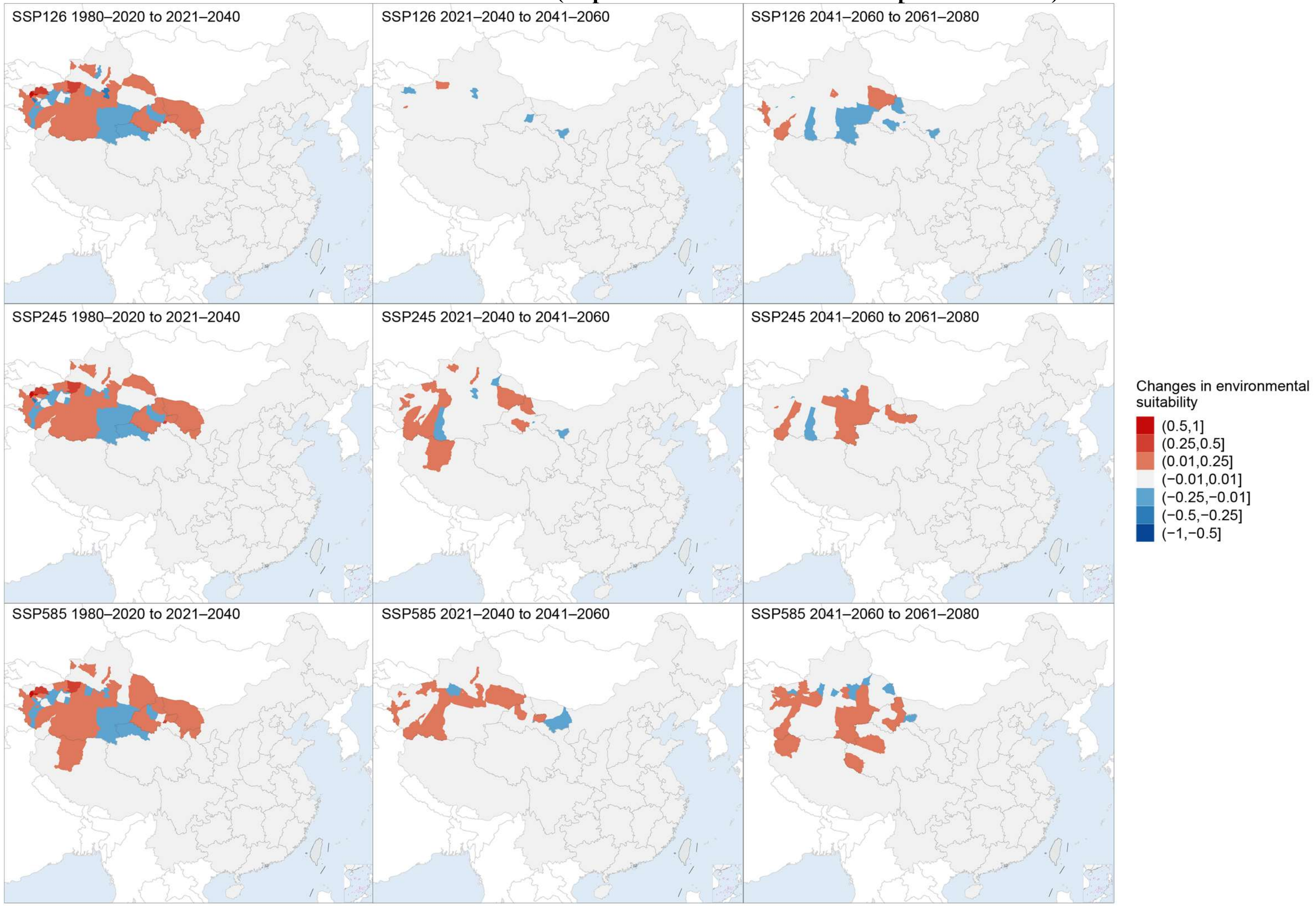

Supplementary Tables

Table A: The specific references for all 47 sandfly species in China from 1940 to 2022.

Sandfly species	References*
C. junlianensis	22, 42, 66, 122, 125-126, 148, 158, 243, 256, 260, 281
I. longiforceps	42, 107, 184, 195, 243, 256, 260, 281
P. alexandri	22, 24, 26, 31, 35, 41-42, 44, 65, 68-69, 81, 84, 92, 101-102, 110, 117, 130, 133, 135, 140, 147, 149-150, 154-156, 166, 169, 179, 197, 199, 221-222, 227, 229, 231, 243, 246, 260, 267, 275, 281-282
P. andrejevi	42, 68, 84, 90, 94, 99-102, 106, 110, 117, 120, 127, 129, 142, 147, 149, 169, 173-174, 179, 183, 227, 243, 260, 264, 267, 281-282
P. caucasicus	42, 84, 101-102, 127, 142, 149, 179, 197, 221, 243, 246, 260, 264, 267, 281
P. chinensis	1, 4-7, 9, 12-13, 17, 22, 26, 29, 31-34, 36, 39-40, 42-43, 48, 50-58, 61-65, 67-70, 73-75, 77-80, 82-83, 85-89, 91, 93, 95, 97-98, 103-105, 108-109, 113-115, 117, 121, 124-126, 128, 130, 134, 136-139, 141, 146, 148, 151-152, 158-162, 165, 167-170, 172, 176-180, 187, 191-193, 195, 201-202, 204-214, 217-223, 225, 227-229, 232-244, 247-251, 253-256, 260-263, 265-276, 279-283
P. fengi	42, 221, 243, 258, 281
P. hoepplii	71, 122, 203, 215, 260, 281
P. kiangsuensis	42, 93, 107, 122, 125-126, 148, 158, 163, 167, 195, 208, 214-215, 221, 234, 236-237, 243, 248, 256, 260, 279, 281
P. lengi	22, 42, 221, 243, 259, 281
P. longiductus	6, 18, 20, 22-24, 26, 32, 35, 37, 41-42, 45-47, 60, 65, 81, 84, 102, 123, 140, 147, 149, 154-156, 179, 221-222, 224, 226-227, 231, 242-243, 260, 275, 281-282
P. mongolensis	42, 48, 52, 65, 68, 84, 90, 94, 99-102, 106, 110, 117, 120, 127, 129, 138, 140, 142, 147, 149, 154-155, 160-161, 166, 169-170, 172-174, 176-179, 187, 191, 193, 198, 206-207, 209, 211-214, 217-218, 221, 227, 233-237, 243, 248, 260, 264, 267, 275, 281-282
P. stantoni	8, 42, 107, 116, 122, 126, 190, 221, 236-237, 241, 243, 260, 281
P. tumenensis	33, 42, 107, 116, 122, 125-126, 132, 148, 158, 188, 195, 200, 221, 243, 256, 260, 279, 281
P. wui	2, 6, 10-11, 15-16, 18-22, 24-26, 28, 30, 32, 35, 37-38, 41-42, 45-47, 49, 59, 65, 68, 81, 84, 96, 101-102, 106, 110-111, 117, 120, 123, 127, 135, 140, 142-144, 147, 149, 154, 156, 164, 169, 173-175, 179, 189, 221-222, 224, 226-227, 231, 242-243, 252, 260, 263-264, 267, 275, 277, 281-282
P. yunshengensis	22, 42, 107, 125-126, 148, 158, 221, 243, 260, 278, 281
S. arpaklensis	42, 84, 90, 101-102, 120, 127, 140, 142, 147, 149, 154-155, 157, 221-222, 229, 243, 260, 264, 281
S. bailyi	3, 8, 14, 42, 107, 116, 118-119, 122, 126, 145, 148, 158, 163, 185-186, 190, 221, 236-237, 241, 243, 256, 260, 279, 281
S. barraudi	33, 42, 71-72, 76, 93, 107-108, 112, 116, 122, 125-126, 131, 148, 153, 158, 167, 171, 186, 188, 208, 210, 215, 221, 236-237, 243, 248, 256, 260, 274, 279, 281
S. fanglianensis	42, 196, 221, 243, 245, 260, 281
S. fukienensis	71, 203, 281
S. fupingensis	48, 179, 217, 236, 281
S. hunanensis	281
S. iyengari	8, 42, 107, 122, 190, 194, 196, 221, 236-237, 241, 243, 245, 260, 281
S. kachekensis	236-237, 281
S. khawi	5, 42, 48, 68, 160, 169, 172, 178-179, 187, 193, 206, 209, 211, 214, 218, 221, 223, 233, 236-237, 243, 249, 260, 281
S. koloshanensis	22, 26, 33, 39, 42, 51, 53, 68, 86, 104, 107, 116, 118-119, 121, 125-126, 148, 152, 158, 167, 169, 179, 186-187, 221, 223, 225, 229, 236, 241, 243, 256, 260, 274, 279, 281
S. kueichenae	107, 221, 281
S. lanzhouensis	42, 221, 243, 281
S. lushanensis	281
S. nankingensis	42, 71-72, 93, 208, 210, 216, 221, 243, 260, 281

S. pooi	236-237, 260, 281
S. quanzhouensis	42, 71, 153, 221, 243, 260, 281
S. rudnicki	42, 107, 221, 243, 260, 281
S. sinkiangensis	16, 38, 42, 49, 81, 84, 101-102, 110, 117, 120, 123, 127, 140, 142, 147, 149, 154-155, 164, 166, 169, 173-175, 179, 189, 221, 231, 243, 252, 260, 264, 267, 275, 281
S. squamipleuris	8, 42, 122, 190, 215, 236-237, 241, 243, 248, 260, 281
S. squamirostris	1, 5, 22, 26-27, 33, 39, 42-43, 48, 50, 53-55, 57-58, 62, 64-65, 68, 73-74, 76, 79-80, 85-86, 91, 93, 98, 104, 108-109, 115, 121, 141, 151-152, 158-159, 169, 171-172, 178-179, 187, 192-193, 201, 206, 208-211, 214, 218, 221, 223, 225, 230, 232-234, 236-237, 241, 243, 248-249, 260, 274, 279, 281
S. sumbarica	84, 101-102, 110, 140, 149, 155, 281
S. suni	5, 33, 42, 48, 65, 68, 104, 121, 138, 145, 152, 171-172, 179, 181-182, 217, 221, 225, 236, 243, 260, 279, 281
S. tangi	42, 221, 243, 281
S. turfanensis	42, 84, 102, 149, 154, 166, 179, 221, 243, 260, 281
S. wangi	221, 257, 281
S. wuyishanensis	71, 153, 221, 260, 281
S. yaoi	260, 281
S. yini	42, 71, 221, 243, 260, 281
S. yunnanensis	42, 221, 243, 260, 281
S. zhongi	42, 221, 243, 260, 281

*See Supplementary References 2.

Table B: The inclusion and exclusion criteria for screening publications

Criteria	Guidance	Outcome
**Title/Abstract screening**		
#1: Sandflies or pathogens	Does the Title/Abstract refer the sandflies and sandfly-associated pathogens?	If Yes, remain and evaluate #2.<br>If No, exclude.
#2: Source of investigation	Does the Title/Abstract refer the sandflies and sandfly-associated pathogens which are from natural environment?	If Yes, remain for full text review.<br>If No, exclude.
**Full text screening**		
#1: Re-screening	Does the article meet the screening criteria before?<br>1-sandflies or sandfly-associated pathogens<br>2-not letters to the editor, opinion and editorial articles, media reports, and abstracts of posters<br>3-collecting or infection in natural environment<br>4-not insecticide, testing tool, drug, or vaccine trials<br>5-not transstadial transmission research in vectors<br>6-not molecular research of sandflies or sandfly-borne pathogens	If Yes, remain and evaluate #2.<br>If No, exclude.
#2: Testing	Does the article refer the specific detection methods of pathogens?<br>1-pathogens-based testing method (e.g., serological, or molecular)<br>2-specific pathogens identified in the detection	If Yes, remain and evaluate #3.<br>If No, exclude from database of pathogens.
#3: Geographical information	Does the article refer the geographical information?<br>1-geographic location information at province, city, or county administrative divisions levels<br>2-exact locations or only marked the latitude and longitude	If Yes, remain for data extracting.<br>If No, exclude.

Table C: Original resolutions and extents of source datasets

Variable	Spatial resolution	Temporal extent	Source of data	Website	References
Climate data	0°2.5′	1980-2018<br>2021–2040<br>2041–2060<br>2061–2080	WorldClim	https://www.worldclim.org/	Fick, S.E. and R.J. Hijmans, 2017. WorldClim 2: new 1km spatial resolution climate surfaces for global land areas. International Journal of Climatology 37 (12): 4302-4315.<br>Harris, I., P.D. Jones, T.J. Osborn, and D.H. Lister (2014), Updated high-resolution grids of monthly climatic observations - the CRU TS3.10 Dataset. International Journal of Climatology 34, 623-642. doi:10.1002/joc.3711
Terrain data	250 m	2000	Resource and Environment Science and Data Center (RESDC)	https://www.resdc.cn/data.aspx?DATAID=123	Institute of Geographic Sciences and Natural Resources Research, Cas. DEM (Digital elevation model) of China. Beijing: Resource and Environment Science and Data Center. (available at: https://www.resdc.cn/data.aspx?DATAID=123)
Socioeconomic data	0.5°	2010–2100	Science Data Bank	https://doi.org/10.57760/sciencedb.01683	Jiang T, Su B, Wang YJ, et al. Gridded datasets for population and economy under Shared Socioeconomic Pathways.

Table D: Potential risk factors at the county level used in the BRT model for sandfly species and 2-stage XGBoost model for VL

Variables	Description	Sandflies	VL	Corresponding References	References
Socioeconomic				[1, 2]	1. Jiang D, Ma T, Hao M, Qian Y,
Rural population proportion	Rural human population proportion (Rural population/Total population)	√	√		Chen S, Meng Z, et al.
Population density	Human population density (persons per km2)	√	√		Spatiotemporal patterns and spatial
Rural population density	Rural human population density (persons per km2)	√	√		risk factors for visceral
Bioclimatic				[1-4]	leishmaniasis from 2007 to 2017 in
Bio1	Annual mean temperature (℃)	√	√		Western and Central China: A
Bio2	Mean diurnal range (℃)	√	√		modelling analysis. Sci Total
Bio3	Isothermality (Mean of monthly max - min temperature) (℃)	√	√		Environ. 2021;764:144275.
Bio4	Temperature seasonality (BIO02 ÷ BIO07 ×100)	√	√		2. Guan LR, Zhou ZB, Jin CF, Fu
Bio5	Max temperature of warmest month (℃)	√	√		Q, Chai JJ. Phlebotomine sand flies
Bio6	Min temperature of coldest month (℃)				(Diptera: Psychodidae) transmitting
Bio7	Temperature annual range (℃)				visceral leishmaniasis and their
Bio8	Mean temperature of wettest quarter (℃)				geographical distribution in China: a
Bio9	Mean Temperature of driest quarter (℃)				review. Infect Dis Poverty.
Bio10	Mean Temperature of warmest quarter (℃)				2016;5:15.
Bio11	Mean temperature of coldest quarter (℃)				3. Cecílio P, Cordeiro-da-Silva A,
Bio12	Total precipitation (mm)	√	√		Oliveira F. Sand flies: Basic
Bio13	Precipitation of wettest month (mm)				information on the vectors of
Bio14	Precipitation of driest month (mm)				leishmaniasis and their interactions
Bio15	Precipitation seasonality (Coefficient of variation)	√	√		with Leishmania parasites. Commun
Bio16	Precipitation of wettest quarter (mm)				Biol. 2022;5(1):305.
Bio17	Precipitation of driest quarter (mm)	√	√		4. Erguler K, Pontiki I, Zittis G,
Bio18	Precipitation of warmest quarter (mm)				Proestos Y, Christodoulou V,
Bio19	Precipitation of coldest quarter (mm)				Tsirigotakis N, et al. A climate-
Terrain				[1, 2, 5]	driven and field data-assimilated
Standard deviation of elevation	Standard deviation of elevation (At county level)	√	√		population dynamics model of sand flies. Sci Rep. 2019;9(1):2469.
Average elevation	Average elevation (m)	√	√		5. Lun ZR, Wu MS, Chen YF,
Sandfly vector				[1-5]	Wang JY, Zhou XN, Liao LF, et al.
Presence of *P. chinensis*	Presence of *P. chinensis* (0/1)		√		Visceral Leishmaniasis in China: an
Presence of *P. wui*	Presence of *P. wui* (0/1)		√		Endemic Disease under Control.
Presence of *P. longiductus*	Presence of *P. longiductus* (0/1)		√		Clin Microbiol Rev.
Presence of *P. alexandri*	Presence of *P. alexandri* (0/1)		√		2015;28(4):987-1004.

Table E: Clustering analysis of model predictors at the county level based on pairwise Pearson correlation coefficients

	bio1 [a]	bio2 [a]	bio3 [a]	bio4 [a]	bio5 [a]	bio6	bio7	bio8	bio9	bio10	bio11	bio12 [a]	bio13	bio14	bio15 [a]	bio16	bio17 [a]	bio18	bio19
bio1						0.951			0.948		0.945								
bio2																			
bio3																			
bio4							0.971												
bio5								0.916		0.979									
bio6									0.985		0.994								
bio7																			
bio8										0.946									
bio9											0.988								
bio10																			
bio11																			
bio12													0.941			0.963		0.870	
bio13																0.992		0.950	
bio14																	0.982		0.942
bio15																			
bio16																		0.953	
bio17																			0.982
bio18																			
bio19																			

Pairwise correlations above 0.8 are shown, and blank off-diagonal cells all have correlations < 0.8. Predictors grouped to the same cluster are colored the same. From each cluster, only one predictor (marked with a) is chosen for models to avoid multicollinearity.

Table F: Cross-tabulation of observed and XGBoost-model-predicted annual incidence levels of VL in 2016

Disease	Observed Incidence Level	Model-Predicted Incidence Level		
		0	1	2
AVL&DT-ZVL	0	2809	15	1
	1	1	26	2
	2	0	0	4
MT-ZVL	0	2649	128	0
	1	2	67	2
	2	0	0	10

Both observed and model-predicted annual incidences were categorized into three levels: 0 ($<0.071/10^5$), 1 ($\geq 0.071/10^5$ but $<1.490/10^5$) and 2 ($\geq 1.490/10^5$). The observed and model-predicted levels were deemed consistent if the most of frequencies occurred in diagonal cells. The presented frequencies are the averages over five models, each with one of the years 2014 to 2018 as the testing set and the remaining years as the training set.

Table G: BRT-model-estimated mean (standard deviation) relative contributions of top factors (RC ≥5%) to the spatial distribution of six most prevalent sandfly species in the *Phlebotomus* genus

Category	Variable	*P. chinensis*	*P. mongolensis*	*P. kiangsuensis*	*P. wui*	*P. longiductus*	*P. alexandri*
Socioeconomic	Rural population proportion		7.01 (1.52)	17.66 (4.35)	5.74 (2.73)	5.43 (3.03)	
	Population density					6.47 (2.79)	
	Rural population density	5.82 (2.89)					
Bioclimatic	Annual mean temperature	29.79 (6.54)	5.23 (1.08)	12.08 (3.83)			
	Mean diurnal range			10.61 (2.85)			11.54 (5.35)
	Isothermality		12.13 (2.55)				
	Temperature seasonality	12.77 (3.20)	31.24 (3.17)	5.15 (2.38)		7.53 (3.22)	
	Max temperature of warmest month		15.93 (3.03)		14.50 (9.75)		
	Total precipitation	22.17 (5.38)	8.89 (1.66)	7.00 (2.34)	48.15 (9.79)	35.78 (6.11)	47.13 (11.95)
	Precipitation seasonality	6.30 (1.82)			5.86 (3.12)	22.59 (6.07)	
	Precipitation of driest quarter			11.84 (2.79)			7.16 (4.35)
Terrain	Standard deviation of elevation		5.67 (1.82)	6.03 (1.89)	8.42 (2.62)	14.35 (3.98)	15.24 (5.05)
	Average elevation		5.49 (1.20)	10.70 (3.02)			6.35 (3.24)
AUC	Train	0.960 (0.949, 0.976)	0.987 (0.977, 0.996)	0.992 (0.984, 0.999)	0.999 (0.998, 1.000)	1.000 (0.998, 1.000)	0.996 (0.989, 1.000)
	Test	0.908 (0.858, 0.946)	0.899 (0.860, 0.934)	0.913 (0.840, 0.970)	0.985 (0.967, 0.998)	0.984 (0.969, 0.997)	0.969 (0.934, 0.990)
Partial AUC ratio	Train	1.33	1.52	1.87	1.90	1.92	1.93
	Test	1.32	1.50	1.87	1.90	1.91	1.90

Mean AUCs (95% percentiles) and partial area AUC ratio (calculated at tolerance level of 0.2) are given.

Table H: BRT-model-estimated mean (standard deviation) relative contributions of top factors (RC ≥5%) to the spatial distribution of six most prevalent sandfly species in the *Sergentomyia* genus

**Category**	**Variable**	***S. squamirostris***	***S. barraudi***	***S. khawi***	***S. koloshanensis***	***S. sinkiangensis***	***S. bailyi***
Socioeconomic	Rural population proportion	7.08 (1.63)	6.35 (2.66)	5.87 (1.65)	18.00 (4.74)		
	Population density			5.59 (2.03)			
Bioclimatic	Annual mean temperature	13.55 (3.83)	7.59 (3.46)	21.97 (3.31)			21.86 (11.04)
	Mean diurnal range	8.90 (2.10)	13.58 (5.96)		6.14 (2.80)		11.36 (5.52)
	Isothermality	8.87 (2.11)	7.59 (2.46)	6.55 (2.05)	10.66 (3.58)	10.45 (4.25)	
	Temperature seasonality	9.98 (2.22)	17.04 (6.32)	5.37 (1.94)	24.42 (4.17)		63.54 (11.14)
	Max temperature of warmest month			9.35 (2.87)	6.39 (2.93)	9.23 (6.10)	
	Total precipitation	14.19 (2.50)	19.22 (5.52)	7.09 (1.65)	6.38 (3.52)	49.00 (10.09)	
	Precipitation seasonality	5.84 (1.86)					
	Precipitation of driest quarter	5.86 (2.74)	6.95 (3.72)			5.45 (3.14)	
Terrain	Standard deviation of elevation	9.70 (1.99)	5.11 (1.83)	16.17 (3.10)	6.80 (2.25)	5.56 (1.95)	
	Average elevation			6.45 (1.88)	7.76 (3.15)		
AUC	Train	0.947 (0.912, 0.979)	0.967 (0.957, 0.988)	0.991 (0.982, 0.997)	0.995 (0.992, 0.999)	0.999 (0.997, 1.000)	0.998 (0.995, 1.000)
	Test	0.810 (0.727, 0.877)	0.906 (0.842, 0.955)	0.868 (0.696, 0.941)	0.941 (0.851, 0.976)	0.982 (0.962, 0.996)	0.982 (0.960, 0.997)
Partial AUC ratio	Train	1.68	1.86	1.89	1.92	1.93	1.93
	Test	1.63	1.74	1.85	1.85	1.92	1.94

Mean AUCs (95% percentiles) and partial area AUC ratio (calculated at tolerance level of 0.2) are given.

Table I: Projections of the numbers, land areas, and population sizes of counties affected by VL risk areas according to SSP126

Province	Number of Counties[#] (Relative difference %)[*]		Area×10³km²[#] (Relative difference %)[*]		Population×10⁴ persons[#] (Relative difference %)[*]	
	High-risk	Low-medium-risk	High-risk	Low-medium-risk	High-risk	Low-medium-risk
AVL&DT-ZVL						
Gansu	–	1/2/1/1/1<br>(100.0/-50.0/0.0/0.0)	–	231.9/494.6/12.6/12.6/12.6<br>(113.3/-97.4/0.0/0.0)	–	15.3/31.8/14.1/13.3/11.5<br>(108.2/-55.7/-5.6/-13.3)
Xinjiang	4/7/12/14/13<br>(75.0/71.4/16.7/-7.1)	28/39/22/21/23<br>(39.3/-43.6/-4.5/9.5)	822.9/2219.3/1360.9/3135.6/3105.3<br>(169.7/-38.7/130.4/-1.0)	5177.6/5878.3/4782.3/3010.0/3253.1<br>(13.5/-18.6/-37.1/8.1)	89.9/115.9/339.1/403.0/379.3<br>(28.9/192.7/18.9/-5.9)	770.5/1203.7/723.8/749.0/871.2<br>(56.2/-39.9/3.5/16.3)
ALL	4/7/12/14/13<br>(75.0/71.4/16.7/-7.1)	29/41/23/22/24<br>(41.4/-43.9/-4.3/9.1)	822.9/2219.3/1360.9/3135.6/3105.3<br>(169.7/-38.7/130.4/-1.0)	5409.5/6373.0/4794.9/3022.6/3265.7<br>(17.8/-24.8/-37.0/8.0)	89.9/115.9/339.1/403.0/379.3<br>(28.9/192.7/18.9/-5.9)	785.8/1235.5/737.9/762.3/882.7<br>(57.2/-40.3/3.3/15.8)
MT-ZVL						
Gansu	4/4/1/0/0<br>(0.0/-75.0/-100.0/0.0)	16/41/0/0/0<br>(156.2/-100.0/0.0/0.0)	155.7/155.7/45.5/0.0/0.0<br>(0.0/-70.8/-100.0/0.0)	517.3/1192.8/0.0/0.0/0.0<br>(130.6/-100.0/0.0/0.0)	111.8/111.8/11.3/0.0/0.0<br>(0.0/-89.9/-100.0/0.0)	353.2/1271.9/0.0/0.0/0.0<br>(260.1/-100.0/0.0/0.0)
Henan	–	3/3/0/0/0<br>(0.0/-100.0/0.0/0.0)	–	19.9/19.9/0.0/0.0/0.0<br>(0.0/-100.0/0.0/0.0)	–	80.7/80.7/0.0/0.0/0.0<br>(0.0/-100.0/0.0/0.0)
Qinghai	–	0/1/0/0/0<br>(-/-100.0/0.0/0.0)	–	0.0/18.6/0.0/0.0/0.0<br>(-/-100.0/0.0/0.0)	–	0.0/20.4/0.0/0.0/0.0<br>(-/-100.0/0.0/0.0)
Shaanxi	–	18/35/5/5/6<br>(94.4/-85.7/0.0/20.0)	–	341.1/758.7/121.4/112.2/121.6<br>(122.5/-84.0/-7.6/8.4)	–	517.8/1079.6/114.4/104.8/107.9<br>(108.5/-89.4/-8.4/2.9)
Shanxi	3/3/2/1/2<br>(0.0/-33.3/-50.0/100.0)	14/39/4/6/7<br>(178.6/-89.7/50.0/16.7)	6.5/6.5/0.7/0.4/0.7<br>(0.0/-89.2/-45.8/84.5)	155.9/447.4/46.5/67.7/83.5<br>(187.0/-89.6/45.7/23.2)	17.7/17.7/2.1/1.4/1.7<br>(0.0/-87.9/-33.8/19.6)	364.3/1169.3/126.2/158.1/207.8<br>(221.0/-89.2/25.2/31.4)
Sichuan	3/5/4/1/0<br>(66.7/-20.0/-75.0/-100.0)	20/32/1/0/0<br>(60.0/-96.9/-100.0/0.0)	138.3/276.5/221.8/54.4/0.0<br>(100.0/-19.8/-75.5/-100.0)	648.9/723.9/38.5/0.0/0.0<br>(11.6/-94.7/-100.0/0.0)	22.7/40.0/32.4/9.6/0.0<br>(76.5/-18.9/-70.4/-100.0)	527.5/1230.4/19.9/0.0/0.0<br>(133.2/-98.4/-100.0/0.0)
Yunnan	–	0/2/0/0/0<br>(-/-100.0/0.0/0.0)	–	0.0/187.3/0.0/0.0/0.0<br>(-/-100.0/0.0/0.0)	–	0.0/30.8/0.0/0.0/0.0<br>(-/-100.0/0.0/0.0)
ALL	10/12/7/2/2<br>(20.0/-41.7/-71.4/0.0)	71/153/10/11/13<br>(115.5/-93.5/10.0/18.2)	300.4/438.6/268.0/54.8/0.7<br>(46.0/-38.9/-79.6/-98.7)	1683.1/3348.7/206.4/179.9/205.1<br>(99.0/-93.8/-12.8/14.0)	152.1/169.5/45.9/11.0/1.7<br>(11.4/-72.9/-76.0/-84.6)	1843.5/4883.0/260.6/262.9/315.6<br>(164.9/-94.7/0.9/20.1)
Total	14/19/19/16/15<br>(35.7/0.0/-15.8/-6.2)	100/194/33/33/37<br>(94.0/-83.0/0.0/12.1)	1123.3/2658.0/1628.8/3190.3/3106.0<br>(136.6/-38.7/95.9/-2.6)	7092.6/9721.6/5001.2/3202.5/3470.7<br>(37.1/-48.6/-36.0/8.4)	242.0/285.3/385.0/414.0/381.0<br>(17.9/34.9/7.5/-8.0)	2629.3/6118.5/998.4/1025.2/1198.3<br>(132.7/-83.7/2.7/16.9)

[#]The number, land area, and population size of counties affected by VL that were actually reported during 2014–2018, projected during 2014–2018, 2021–2040, 2041–2060, and 2061–2080, with values for different time periods separated by slashes.

[*]The relative differences (%) was estimated for the predicted values as compared to those of the previous period, i.e., from actual observations to projection during 2014–2018, from the projection during 2014–2018 to 2021–2040, from the projection during 2021–2040 to 2041–2060, and from the projection during 2041–2060 to 2061–2080, that were intermitted by slash in the parentheses.

"–" indicates that the predicted values for the counties, areas, or population size affected by VL cases are zero for all periods.

VL visceral leishmaniasis; SSP Shared Socioeconomic Pathway; AVL&DT-ZVL anthroponotic VL and desert-type zoonotic VL; MT-ZVL mountain-type zoonotic VL.

Table J: Projections of the numbers, land areas, and population sizes of counties affected by VL risk areas according to SSP245

Province	Number of counties# (Relative difference %)*		Area×10^3km^2# (Relative difference %)*		Population×10^4 persons# (Relative difference %)*	
	High-risk	Low-medium-risk	High-risk	Low-medium-risk	High-risk	Low-medium-risk
AVL&DT-ZVL						
Gansu	–	1/2/1/1/2<br>(100.0/-50.0/0.0/100.0)	–	231.9/494.6/12.6/12.6/275.4<br>(113.3/-97.4/0.0/2082.5)	–	15.3/31.8/14.5/14.2/29.2<br>(108.2/-54.4/-2.1/106.0)
Xinjiang	4/7/13/12/14<br>(75.0/85.7/-7.7/16.7)	28/39/20/24/28<br>(39.3/-48.7/20.0/16.7)	822.9/2219.3/2742.5/1360.9/1385.3<br>(169.7/23.6/-50.4/1.8)	5177.6/5878.3/3006.3/4920.0/5456.5<br>(13.5/-48.9/63.7/10.9)	89.9/115.9/359.5/395.6/451.8<br>(28.9/210.3/10.0/14.2)	770.5/1203.7/710.3/887.0/1072.1<br>(56.2/-41.0/24.9/20.9)
All	4/7/13/12/14<br>(75.0/85.7/-7.7/16.7)	29/41/21/25/30<br>(41.4/-48.8/19.0/20.0)	822.9/2219.3/2742.5/1360.9/1385.3<br>(169.7/23.6/-50.4/1.8)	5409.5/6373.0/3018.9/4932.6/5731.8<br>(17.8/-52.6/63.4/16.2)	89.9/115.9/359.5/395.6/451.8<br>(28.9/210.3/10.0/14.2)	785.8/1235.5/724.8/901.1/1101.3<br>(57.2/-41.3/24.3/22.2)
MT-ZVL						
Gansu	4/4/0/0/0<br>(0.0/-100.0/0.0/0.0)	16/41/0/0/1<br>(156.2/-100.0/0.0/-)	155.7/155.7/0.0/0.0/0.0<br>(0.0/-100.0/0.0/0.0)	517.3/1192.8/0.0/0.0/95.0<br>(130.6/-100.0/0.0/-)	111.8/111.8/0.0/0.0/0.0<br>(0.0/-100.0/0.0/0.0)	353.2/1271.9/0.0/0.0/46.1<br>(260.1/-100.0/0.0/-)
Henan	–	3/3/0/0/0<br>(0.0/-100.0/0.0/0.0)	–	19.9/19.9/0.0/0.0/0.0<br>(0.0/-100.0/0.0/0.0)	–	80.7/80.7/0.0/0.0/0.0<br>(0.0/-100.0/0.0/0.0)
Qinghai	–	0/1/0/0/0<br>(-/-100.0/0.0/0.0)	–	0.0/18.6/0.0/0.0/0.0<br>(-/-100.0/0.0/0.0)	–	0.0/20.4/0.0/0.0/0.0<br>(-/-100.0/0.0/0.0)
Shannxi	–	18/35/4/6/7<br>(94.4/-88.6/50.0/16.7)	–	341.1/758.7/96.7/138.6/169.0<br>(122.5/-87.3/43.3/22.0)	–	517.8/1079.6/84.2/157.4/156.6<br>(108.5/-92.2/86.8/-0.5)
Shanxi	3/3/2/2/2<br>(0.0/-33.3/0.0/0.0)	14/39/5/8/7<br>(178.6/-87.2/60.0/-12.5)	6.5/6.5/0.7/0.7/0.7<br>(0.0/-89.2/0.0/0.0)	155.9/447.4/51.2/92.2/90.2<br>(187.0/-88.6/80.2/-2.2)	17.7/17.7/2.2/2.1/1.9<br>(0.0/-87.6/-4.6/-10.9)	364.3/1169.3/147.2/216.8/214.0<br>(221.0/-87.4/47.3/-1.3)
Sichuan	3/5/4/1/1<br>(66.7/-20.0/-75.0/0.0)	20/32/1/0/0<br>(60.0/-96.9/-100.0/0.0)	138.3/276.5/221.8/54.4/54.4<br>(100.0/-19.8/-75.5/0.0)	648.9/723.9/38.5/0.0/0.0<br>(11.6/-94.7/-100.0/0.0)	22.7/40.0/33.2/10.1/8.6<br>(76.5/-17.0/-69.6/-14.4)	527.5/1230.4/20.4/0.0/0.0<br>(133.2/-98.3/-100.0/0.0)
Yunnan	–	0/2/0/0/0<br>(-/-100.0/0.0/0.0)	–	0.0/187.3/0.0/0.0/0.0<br>(-/-100.0/0.0/0.0)	–	0.0/30.8/0.0/0.0/0.0<br>(-/-100.0/0.0/0.0)
All	10/12/6/3/3<br>(20.0/-50.0/-50.0/0.0)	71/153/10/14/15<br>(115.5/-93.5/40.0/7.1)	300.4/438.6/222.5/55.1/55.1<br>(46.0/-49.3/-75.2/0.0)	1683.1/3348.7/186.3/230.7/354.2<br>(99.0/-94.4/23.8/53.5)	152.1/169.5/35.3/12.1/10.5<br>(11.4/-79.1/-65.6/-13.8)	1843.5/4883.0/251.8/374.2/416.7<br>(164.9/-94.8/48.6/11.4)
Total	14/19/19/15/17<br>(35.7/0.0/-21.1/13.3)	100/194/31/39/45<br>(94.0/-84.0/25.8/15.4)	1123.3/2658.0/2965.0/1416.0/1440.5<br>(136.6/11.6/-52.2/1.7)	7092.6/9721.6/3205.3/5163.3/6086.0<br>(37.1/-67.0/61.1/17.9)	242.0/285.3/394.8/407.7/462.2<br>(17.9/38.4/3.3/13.4)	2629.3/6118.5/976.6/1275.3/1518.0<br>(132.7/-84.0/30.6/19.0)

#The number, land area, and population size of counties affected by VL that were actually reported during 2014–2018, projected during 2014–2018, 2021–2040, 2041–2060, and 2061–2080, with values for different time periods separated by slashes.

*The relative differences (%) was estimated for the predicted values as compared to those of the previous period, i.e., from actual observations to projection during 2014–2018, from the projection during 2014–2018 to 2021–2040, from the projection during 2021–2040 to 2041–2060, and from the projection during 2041–2060 to 2061–2080, that were intermitted by slash in the parentheses.

"–" indicates that the predicted values for the counties, areas, or population size affected by VL cases are zero for all periods.

VL visceral leishmaniasis; SSP Shared Socioeconomic Pathway; AVL&DT-ZVL anthroponotic VL and desert-type zoonotic VL; MT-ZVL mountain-type zoonotic VL.

Supplementary References 2

19 Chen H. M. et al., Investigation of vector microhabitats in leishmaniasis endemic areas in the extended Loess Plateau of China. Chinese Journal of Vector Biology and Control **30** 597 (2019).

20 Han S. et al., Analysis of visceral leishmaniasis epidemic in China, 2004–2016. Chinese Journal of Parasitology and Parasitic Diseases **37** 189 (2019).

21 Peng H. et al., Survey of important vectors in Sansha, China. China Tropical Medicine **19** 128 (2019).

22 Bai X. F. et al., Investigation on the familial aggregation and spatial aggregation of people infected with leishmania in Tanchang County of Gansu Province. Journal of Preventive Medicine Information **34** 1502 (2018).

23 Wang F. P. et al., Epidemiological analysis of visceral leishmaniasis in Hancheng City of Shaanxi Province in 2017. Chinese Journal of Parasitology and Parasitic Diseases **36** 483 (2018).

24 Guan L. R. & Gao C. H., Leishmaniasis and its control. Chinese Journal of Parasitology and Parasitic Diseases **36** 418 (2018).

25 Wang F. P. et al., Characteristics and risk factors of the kala-azar in Hancheng City, Shaanxi Province, China. Chinese Journal of Zoonoses **34** 756 (2018).

26 Chen H. Y. et al., Molecular characteristics of common sandflies species (Diptera: Psychodidae) in Hainan Province. Chinese Journal of Vector Biology and Control **29** 15 (2018).

27 Tang Z. Q., Gao P., Zhou K. H., Liu J. Q. & Guo X. S., A survey on sandfly in Linzhou City of Henan Province. Chinese Journal of Hygienic Insecticides & Equipments **23** 397 (2017).

28 Liao L. F., Liu Z. J., Adili S., Cui Y. & Xu Y. M., The observation and analysis of impact on desert-type kala-azar by aerial control of Apocheima cinerarius in populus euphratica. Chinese Journal of Vector Biology and Control **28** 478 (2017).

29 Yisilayin W. et al., Surveillance of sand flies at desert-like area in Jiashi County of Xinjiang: potential vectors of visceral leishmaniasis. Chinese Journal of Parasitology and Parasitic Diseases **35** 194 (2017).

30 Zhang L. & Ma Y. J., Molecular population genetic structure of Phebotomus chinensis (Diptera: Psychodidae) in China inferred by mitochondrial DNA. China Tropical Medicine **16** 947 (2016).

31 Ban Y. P. & Jing Q., Prevalence trend of kala-azar disease in Lixian County of Sichuan Province, 1964–2015. Parasitoses and Infectious Diseases **14** 179 (2016).

32 Yang M. L. et al., Population dynamics of the blood-sucking insects and detection of pathogens in the rodents from Xisha Islands of China. Academic Journal of Second Military Medical University **37** 355 (2016).

33 Aideer A. A. et al., Seroepidemiological survey and analysis of kala-azar in Minfeng County of Xinjiang. Bulletin of Disease Control & Prevention (China) **30** 1 (2015).

34 Aideer A. A. et al., First isolation of leishmania from Phlebotomus wui in Minfeng County, Xinjiang. Bulletin of Disease Control & Prevention (China) **30** 15 (2015).

35 Yu D. W., Ding G. W., Ge P. F., Feng Y. & Li F., Retrospective analysis of the prevalence of visceral leishmaniasis in Gansu Province from 2005–2014. Chinese Journal of Parasitology and Parasitic Diseases **33** 208 (2015).

36 Zhao G. H. et al., Risk factors of kala-azar outbreak in Kashgar Prefecture, Xinjiang, China. Chinese Journal of Zoonoses **31** 592 (2015).

37 Chen K. et al., Investigation of infection with leishmania infantum in humans in Jiashi County, Xinjiang. Journal of Pathogen Biology **10** 261 (2015).

38 Zhou Z. B. et al., Investigation on fluctuation of main sandfly species composition ratio and its relationship with visceral leishmaniasis in kaxgar oasis, Xinjiang. International Journal of Medical Parasitic Diseases **42** 82 (2015).

39 Liao L. F., Wu S. B., Zhang J. M. & Yan S. S., Investigation on natural host animals of desert type of kala-azar in Tarim Basin. Chinese Journal of Vector Biology and Control **26** 151 (2015).

40 Zhou Z. B. et al., Species identification of partial sandflies from China with DNA barcoding. Chinese Journal of Zoonoses **30** 1209 (2014).

41 De Ping Wu et al., Analysis of kala-azar surveillance results in Shufu County, Xinjiang, 2005–2012. Journal of Tropical Medicine **14** 1095 (2014).

42 Zhou Z. B., Zhang Y., Zhu H. M., Shi W. Q. & Jin C. F., Identification of three common sandfies in southern Xinjiang with multiplex PCR. Chinese Journal of Parasitology and Parasitic Diseases **32** 185 (2014).

43 Zhang C. Y. et al., Analysis on the genetic polymorphism of microsatellite markers in leishmania donovani isolates from different epidemic foci. Modern Preventive Medicine **41** 1852 (2014).

44 Zhou Z. B. et al., Typing 6 common sandflies species from endemic areas of leishmaniasis in China by PCR-RFLP method. Chinese Journal of Zoonoses **30** 448 (2014).

45 Guan L. R., Prospect on the investigation of sandflies Diptera Psychodidae) in China. Chinese Journal of Parasitology and Parasitic Diseases **31** 310 (2013).
46 Huang C. Z. et al., Case-control study on the influencing factors of kala-azar in the foci of desert-type kala-azar. Chinese Journal of Zoonoses **29** 775 (2013).
47 Jing Q., Analysis of prevalence of kala-azar in Aba Prefecture from year 1958 to 2010. Modern Preventive Medicine **40** 2349 (2013).
48 Yisilayin W., Tong S. X. & Hou Y. Y., Investigation on epidemic outbreak of visceral leishmaniasis in Jiashi County, Xinjiang in 2008. Bulletin of Disease Control & Prevention (China) **27** 1 (2012).
49 Gu D. A., Zhang Y. & Lan Q. X., Analysis of polymorphism on ribosomal internal transcribed spacer 2 in four species of sandflies with PCR-RFLP. Chinese Journal of Parasitology and Parasitic Diseases **30** 100 (2012).
50 Zhang L. & Ma Y. J., Identification of Phlebotomus chinensis (Diptera: Psychodidae) inferred by morphological characters and molecular markers. Entomotaxonomia **34** 71 (2012).
51 He G. B., Tian F. Y. & Zhang F. N., Prevalent trend and control strategies of leishmaniasis in Maoxian County of Sichuan Province. Journal of Preventive Medicine Information **28** 89 (2012).
52 Peng W. P. et al., Survey on leishmania infection in dogs in Sichuan epidemic area in 2010. Veterinary Science in China **42** 93 (2012).
53 Yisilayin W. et al., Surveillance of intermediary sandflies of visceral leishmaniasis in different epidemic areas of southern Xinjiang. Bulletin of Disease Control & Prevention (China) **26** 21 (2011).
54 Zhang J. G., Zhang F. N. & Chen J. P., Prevalence and control situation of canine borne kala azar in Sichuan Province. Journal of Preventive Medicine Information **27** 869 (2011).
55 Yisilayin W. & Hou Y. Y., Retrospective analysis of prevalence of visceral leishmaniasis in Xinjiang from 2005 to 2010. Bulletin of Disease Control & Prevention (China) **26** 3 (2011).
56 Gu D. A. et al., Investigation of visceral leishmaniasis vector sand flies in Gashi County, Xinjiang. Chinese Journal of Parasitology and Parasitic Diseases **28** 280 (2010).
57 Li Y. et al., New trends in the development of kala-azar epidemic and countermeasures in Wudu District, Longnan City, Gansu. Bulletin of Disease Control & Prevention (China) **25** 38 (2010).
58 Zhang L., Fan Y. & Ma Y. J., Isolation of microsatellite DNA and the polymorphic locus screening from Phlebotomus chinensis (Diptera Psychodidae). Chinese Journal of Parasitology and Parasitic Diseases **27** 503 (2009).
59 Zuo X. P. et al., Study of the prevalence of different forms of kala-azar in southern regions of Xinjiang. Journal of Pathogen Biology **4** 960 (2009).
60 Sun J. M., Zhang L. M. & Xu F., A numerical taxonomic study of (Diptera: Psychodidae) from China. Acta Entomologica Sinica **52** 1356 (2009).
61 Hong J. & Zhang H. W., Fengcheng medical insects known list. Journal of Medical Pest Control **25** 512 (2009).
62 Yisilayin W. et al., An epidemiological survey of visceral leishmaniasis in Hamangou coal mine area of Korla City, Xinjiang. Chinese Journal of Parasitology and Parasitic Diseases **27** 237 (2009).
63 Zuo X. P., Mai M. T., A L. M., Zhang S. & Kai S. E., Investigation report of kala-azar in Shufu and Wushi counties of Xinjiang in 2003. Bulletin of Disease Control & Prevention (China) **24** 56 (2009).
64 Meng B. L. et al., A survey on the current situation of kala-azar epidemic in Kashgar, Xinjiang from 2004 to 2006. Bulletin of Disease Control & Prevention (China) **24** 69 (2009).
65 Kai S. E. et al., Reflections on the prevention and control of kala-azar in Kashgar, Xinjiang. Bulletin of Disease Control & Prevention (China) 23 (2008).
66 Yang Y. S., Zhang J. J., Diwu J. X. & Gao X. L., Species and distribution of sand flies in Shaanxi, Qinghai and Ningxia Provinces. Chinese Journal of Hygienic Insecticides & Equipments 255 (2008).
67 Jin C. F. et al., A newly identified endemic area of visceral leishmaniasis in Minfeng County of south Xinjiang ii. investigation on Phlebotomine vectors. Chinese Journal of Parasitology and Parasitic Diseases 132 (2008).
68 Luo J. C., Sun W. K. & Wu Y. L., Observation on the effect of spraying baythroid in animals vault in preventing and controlling the medi-um of leishmaniasis-Phlebotomus chinensis. Journal of Pathogen Biology 403 (2007).
69 Gu D. A. et al., Preliminary experiments on the monitoring of sand fly by light trapping method. Chinese Journal of Parasitology and Parasitic Diseases 160 (2007).
70 Chen S. Y., Analysis of the epidemiological dynamics of kala-azar in Huan County from 1984 to

2005. Modern Preventive Medicine 1166 (2007).
71 Wang J., Guan L. R. & Liu P. Z., Description of the ecological habits of sergentomyia koloshanensis. Chinese Journal of Parasitology and Parasitic Diseases 77 (2007).
72 Yan Q. Y., He L. J., Luo J. C., Li D. F. & Le Yuan Shang, Effectiveness of fluthrin spraying in livestock kilns for the control of Phlebotomus chinensis. Chinese Journal of Vector Biology and Control 419 (2006).
73 Luo J. C. & Zhang K. B., Survey of kala-azar vector sand flies in Shan County, Henan Province. Journal of Pathogen Biology 109 (2006).
74 Tang Z. M., Li G. R., Wang S. G., Mu Z. M. & Luo Q., Investigation and control of kala-azar epidemic in Beichuan County. Journal of Preventive Medicine Information 71 (2006).
75 Xu B. L. et al., Survey and analysis of the prevalence of important human parasitic diseases in Henan Province. Journal of Pathogen Biology 454 (2005).
76 Luo J. C. & Zhao N. H., Survey of kala-azar vector sand flies in Shan County, Sanmenxia. Henan Journal of Preventive Medicine 287 (2005).
77 Liao L. F. et al., Isolation of a Leishmania strain from infants with kala-azar in Ba Chu County, Xinjiang, 2005. Bulletin of Disease Control & Prevention (China) 7 (2005).
78 Zuo X. P., Deng Z. B., A L. M. & Zhang S., Survey on the current situation of kala-azar (visceral leishmaniasis) in Usher County, Xinjiang. Bulletin of Disease Control & Prevention (China) 33 (2005).
79 Li G. R. et al., Survey on the current status of kala-azar epidemic in Heishui County, Sichuan Province. South China Journal of Preventive Medicine 18 (2004).
80 Yan Q. Y. et al., A survey on the residual situation of kala-azar vector sand flies in Henan Province. Journal of Pathogen Biology 62 (2004).
81 De Ming Li, Chen Y., Wei L. S. & Si E. C., Kala-azar prevalence and control strategies in Longnan, Gansu Province. Bulletin of Disease Control & Prevention (China) 95 (2004).
82 Shi S. Z., Liu Z. J., Zhang J. J. & Gong Z. W., Investigation and study of important vector organisms in the Tianshui area. Chinese Journal of Vector Biology and Control 397 (2004).
83 Guo D. X., Jin C. F., Hong Y. M., Ni B. & Qiao Z. D., Ultrastructural study on pharyngeal armatures of seven species of sandflies in China by scanning electron microscopy. Chinese Journal of Parasitology and Parasitic Diseases 37 (2004).
84 Zhang L. M. & Leng Y. J., Scanning electron microscopic observation of Chinius junlianensis leng (1987) and its evolutionary relationship with insects of the subfamily Sand fly. Journal of Pathogen Biology 20 (2004).
85 Li G. R. et al., Epidemiological investigation of kala-azar in Beichuan County, Sichuan Province. Parasitoses and Infectious Diseases 180 (2003).
86 Chen S. B. et al., The sand fly system of Gansu Province. Chinese Journal of Parasitology and Parasitic Diseases 58 (2003).
87 Xiong G. H. & Jin C. F., Research on Sand fly and Leishmaniasis and the Development of Western China. Chinese Journal of Parasitology and Parasitic Diseases 57 (2003).
88 Tang Z. L. et al., Current situation of kala-azar prevalence and control in Heishui County, Sichuan Province. Parasitoses and Infectious Diseases 111 (2002).
89 Xu B. H., The investigation of the medical insects in Fujian Province vi (Diptera: Psychodidae). Journal of Medical Pest Control 246 (2002).
90 Xiong G. H. & Jin C. F., Study on the morphology of the nail of the Sergentomyia nankingensis. Chinese Journal of Parasitology and Parasitic Diseases 44 (2001).
91 Ren B., Qingdao area medical insect animal list. Chinese Journal of Vector Biology and Control 303 (2001).
92 Yan Q. Y. & Le Yuan Shang, Kala-azar surveillance in Henan Province 1983–1999. Parasitoses and Infectious Diseases 81 (2001).
93 Li G. R. & Gao B., Kala-azar of hill type in the western part of China. Parasitoses and Infectious Diseases 29 (2001).
94 Zuo S. L. et al., Surveillance of Phlebotomine sandflies in Hubei Province. Chinese Journal of Zoonoses 107 (2001).
95 Zhang W. H. et al., Observation of Phlebotomus chinensis on seasonal distribution and ecological habits in Heishui County, Sichuan Province. Bulletin of Disease Control & Prevention (China) 37 (2000).
96 Guan L. R., Qu J. Q. & Chai J. J., Leishmaniasis in China-present status of prevalence and some suggestions on its control. Bulletin of Disease Control & Prevention (China) 49 (2000).
97 Gao B. et al., Epidemiological investigation report of kala-azar in Sichuan Province. Journal of Preventive Medicine Information 142 (2000).

98 Hong J., Kong Q. A., Zhang B. G. & Jing R. X., List of blood-sucking arthropods in Fengcheng City, Liaoning Province. Journal of Medical Pest Control 53 (2000).
99 Guan L. R., Chai J. J. & Zuo X. P., Advances in the biology of sand fly in Xinjiang. Bulletin of Disease Control & Prevention (China) 87 (1999).
100 Chen S. B., Li F., He J. P. & Liu P. Z., Monitoring of Sand flies in Qinan County, Gansu Province. Chinese Journal of Parasitology and Parasitic Diseases 22 (1999).
101 Zhao J. H., Qiao Z. D., Yin G. R., Yin L. & Li G. J., The effect of random amplified polymorphic DNA of Phlebotomus chinensis by geographical position. Chinese Journal of Zoonoses 27 (1998).
102 Zuo X. P., Chai J. J. & Guan L. R., A study on sand flies in Xinjiang Uygur Autonomous Region in the past forty years. Bulletin of Disease Control & Prevention (China) 97 (1998).
103 Yan Q. Y., Le Yuan Shang, He L. J. & Li D. F., Survey of kala-azar vector sand flies in Henan. Chinese Journal of Vector Biology and Control 22 (1998).
104 Lu D. M. et al., Epidemiological investigation of kala-azar in Wen County, Gansu Province. Parasitoses and Infectious Diseases 15 (1998).
105 Li G. R., Wu Y. X., Gao B., Wang H. Q. & He S. Y., Epidemiological modal survey of kala-azar in Qingchuan County (surrounding counties in the endemic area), Sichuan Province. Bulletin of Disease Control & Prevention (China) 105 (1998).
106 Yan Q. Y., He L. J., Le Yuan Shang, Song J. D. & Li D. F., Kala-azar surveillance report in Henan Province from 1989 to 1996. Journal of Pathogen Biology 78 (1998).
107 Li G. R., Wu Y. X., Gao B., He S. Y. & Wang H. Q., Phlebotomus chinensis found in Qingchuan County, Sichuan Province. Sichuan Journal of Zoology 35 (1998).
108 Guan L. R., Xu Y. X., Zuo X. P. & Wang G., Sandfly fauna in 'wind-eroded castle' region in Wuerhe of Xinjiang. Bulletin of Disease Control & Prevention (China) 39 (1997).
109 Song J. D., Ding S. Z. & Zhu Y. X., China's kala-azar control status and countermeasures. Henan Journal of Preventive Medicine 172 (1997).
110 Guan L. R., Studies on Phlebotomus (para Phlebotomus) alexandri (sinton, 1928) in China. Bulletin of Disease Control & Prevention (China) 109 (1997).
111 Gui A. F., Le Qun Hu, Zuo S. L., Wu C. X. & Gui X. E., Review and current status of the epidemic and control of kala-azar in Hubei Province. Journal of Pathogen Biology 77 (1997).
112 Guan L. R., Yang Y. Q., Xu Y. X., Qu J. Q. & Zuo X. P., The discovery and study of Leishmania turanica in China. Journal of Medical Research 28 (1997).
113 Chen S. B., Chen X. W., He J. P., Yang X. D. & Yang H. P., A new outbreak area of kala-azar in Gansu Province, Diebu County. Chinese Journal of Parasitology and Parasitic Diseases 31 (1997).
114 Yang Y. Q., Guan L. R. & Wu J. T., Study on the parasitic characteristics of Leishmania infantum in monkeys in the Karamay region. Bulletin of Disease Control & Prevention (China) 8 (1997).
115 Wu Y. X., Li G. R., Gao B. & Di Liu C., Preliminary investigation report of kala-azar in Beichuan County, Sichuan Province. Bulletin of Disease Control & Prevention (China) 43 (1996).
116 Xue S. Q. et al., Sand fly density monitoring report in Beijing. Chinese Journal of Vector Biology and Control 403 (1996).
117 Yang Y. Q., Guan L. R. & Wu J. T., Study on the pathogenicity of Leishmania spp. in Qitai and Fukang, Xinjiang. Bulletin of Disease Control & Prevention (China) 19 (1996).
118 Xu Y. X., Guan L. R., Bao Y. F. & Qu J. Q., Leishmania donovani continues to be detected in the desert areas of northern Xinjiang. Chinese Journal of Parasitology and Parasitic Diseases 71 (1996).
119 Guan L. R., Xu Y. X., Zuo X. P. & Wang G., Study on the vectors of cutaneous leishmaniasis in Karamay, Xinjiang. Bulletin of Disease Control & Prevention (China) 38 (1996).
120 Guan L. R., Chai J. J. & Yang L. P., Geographical distribution of sand flies in Xinjiang Uygur Autonomous Region in relation to geographical landscape. Chinese Journal of Parasitology and Parasitic Diseases 28 (1996).
121 Wu Y. X., Li G. R., Liao Q. Y., Cheng Y. F. & Gao B., Study on the relationship between leishmaniasis and the natural environment of the endemic area. Sichuan Environment 38 (1995).
122 Jin C. F. et al., The ecology of Phlebotomus chinensis in caves of northern Sichuan and its relationship with control. Chinese Journal of Parasitology and Parasitic Diseases 35 (1995).
123 Wu Y. X., Liao P. Y., Li G. R. & Gao B., Current status of kala-azar epidemic in Sichuan Province and progress of research on its prevention and control. Parasitoses and Infectious Diseases 81 (1995).
124 Guan L. R. & Chai J. J., Leishmania protozoa and its relationship with cutaneous leishmaniasis in the Karamay region of Xinjiang. Bulletin of Disease Control & Prevention (China) 46 (1995).
125 Leng Y. J. & He M. S., A study of Phlebotomine sandflies in Yunan Province. Sergentomyia neophlebotomus kuekchenae sp.nov. (Diptera: Psychodidae). Insect Science 13 (1995).

126 Wu Y. X., Li G. R., Gao B. & Tang Z. M., Phlebotomus chinensis found in Beichuan County, Sichuan Province. Sichuan Journal of Zoology 6 (1995).
127 Xue S. Q., Han Y. H., Ceng X. W., De Sheng Zheng & Ren S. L., Preliminary report on monitoring the density of sand fly in Beijing area. Chinese Journal of Vector Biology and Control 65 (1995).
128 Guan L. R., Xu Y. X., Zuo X. P., Zhang S. & Chai J. J., A survey on the survival environment of giant gerbils in northern Xinjiang and inter-rodent leishmania protozoa and vectors. Bulletin of Disease Control & Prevention (China) 7 (1994).
129 Guan L. R. et al., Leishmaniasis XIV in the Karamay region. Identification of the flagellomes of the Phlebotomus major wui subspecies before natural infection. Chinese Journal of Parasitology and Parasitic Diseases 257 (1994).
130 Lu T. M., Liu Y. X., Lin Q. Z. & Li D. P., Observations on the biological traits of the Sergentomyia barraudi. Bulletin of Disease Control & Prevention (China) 35 (1994).
131 Leng Y. J. & Zhang L. M., Phlebotomus sichuanensis in the High Mountain Region of Southwest China. Sichuan Journal of Zoology 9 (1994).
132 Wei L. S., Ren W. W. & Liu P. Z., Survey on the current situation of kala-azar epidemic and epidemic factors in Longnan, Gansu Province. Bulletin of Disease Control & Prevention (China) 69 (1993).
133 Yan Q. Y. et al., 1991–1992 Henan Province kala-azar surveillance survey report. Henan Journal of Preventive Medicine 234 (1993).
134 Lu T. M., Shu G. H., Yue J. H., Cheng Z. L. & Lin Q. Z., A study of the Sergentomyia barraudi in Yibin, Sichuan. Bulletin of Disease Control & Prevention (China) 41 (1993).
135 Guan L. R., Xu Y. X., Jin C. F., Huo S. L. & Kang M. X., A survey on the distribution of sand fly species in Inner Mongolia. Parasitoses and Infectious Diseases 34 (1993).
136 Xiang B. C., Wen X. M. & Yan J. C., Preliminary ecological observations of the Sergentomyia bailyi and Sergentomyia koloshanensis. Sichuan Journal of Zoology 26 (1992).
137 Xiang B. C., Wen X. M. & Yan J. C., Preliminary observations on the hemophilic habits of two species of sand flies in Yibin area. Journal of Medical Pest Control 219 (1992).
138 Guan L. R. et al., Leishmaniasis in the Karamay region IX. Identification of flagellomes before natural infection of sand flies. Bulletin of Disease Control & Prevention (China) 9 (1992).
139 Xiong G. H. et al., Study on the biology of Phlebotomus chinensis and its relationship with Visceral leishmaniasis in humans and dogs in Longnan and Chuanbei. Wuyi Science Journal 7 (1992).
140 Zhang L. M., Ge N. L., Wang H. B. & Leng Y. J., Sand flies of Guangdong Province and their geographical distribution. Journal of Medical Pest Control 19 (1992).
141 Wang G. et al., Further study on the ecology of sand flies in the oasis region of Kashgar, Xinjiang. Bulletin of Disease Control & Prevention (China) 72 (1992).
142 Wu Y. X. et al., Epidemiological investigation of kala-azar in Wenchuan County, Sichuan Province. Bulletin of Disease Control & Prevention (China) 59 (1991).
143 Xiang B. C., Wen X. M., Yan J. C., Leng Y. J. & Tang Z. W., Preliminary observations on the haemophilic habits, life history and overwintering of Sergentomyia barraudi in Yibin area. Sichuan Journal of Zoology 15 (1991).
144 Lu T. M., Preliminary survey of sand fly species in Yibin area, Sichuan Province. Chinese Journal of Parasitology and Parasitic Diseases 31 (1991).
145 Guan L. R. et al., Leishmaniasis in the Karamay region - V. Investigation and study of the ecology of sand fly. Bulletin of Disease Control & Prevention (China) 55 (1991).
146 You X. C., Song J. D., Yan Q. Y. & Zhong Y. G., Henan Province in 1990 kala-azar surveillance report. Henan Journal of Preventive Medicine 696 (1991).
147 Shen B. G., Guan L. R. & Xu Y. X., Leishmaniasis in the Karamay area - IV. Submicroscopic structure of the flagellum-free body of a naturally infected preflagellate of the sand fly inoculated with mice. Bulletin of Disease Control & Prevention (China) 35 (1991).
148 Qu J. Q., Xu Y. X., Guan L. R. & Bao Y. F., Application of monoclonal antibodies for the detection of pre-flagellar bodies in sand flies. Chinese Journal of Parasitology and Parasitic Diseases 36 (1990).
149 Lu T. M. & Shu G. H., Preliminary observations on the survival life span and spawning of the Sergentomyia barraudi. Sichuan Journal of Zoology 21 (1990).
150 Ding S. D., Cai Y. L. & Tao R. H., A discussion on the morphology of the larvae of the Phlebotomus tumenensis and its taxonomy. Journal of Nanjing Medical University (Natural Sciences) 295 (1990).
151 Jia J. X., Guan L. R., Xu Y. X., Wang G. & Hao K. F., A study on the effect of five repellents on

Phlebotomus alexandri. Chinese Journal of Parasitology and Parasitic Diseases 45 (1990).
152 Qiao Z. D. & Lu Y. L., Scanning electron microscope observation of the body structure of adult Phlebotomus chinensis. Bulletin of Disease Control & Prevention (China) 52 (1990).
153 Guan L. R., Xu Y. X., Wang G. & Jia J. X., Observations on the infectivity of Leishmania dubliniensis on Alexandrium lacewing in different regions of China. Chinese Journal of Parasitology and Parasitic Diseases 26 (1990).
154 Zhang L. M., Observations on the captive breeding of Sichuan lacewing and its development in the laboratory. Acta Entomologica Sinica 380 (1990).
155 Li Y. M. & Guan L. R., Investigation report of kala-azar in the mountains of Longnan and northern Sichuan. Journal of Pathogen Biology 13 (1990).
156 Qiao Z. D. & Lu Y. L., Scanning electron microscopic study of five common sand fly pharyngeal nail species in China. Bulletin of Disease Control & Prevention (China) 67 (1989).
157 Cui S. Z., Li T. X., Zhang J. P., Li H. M. & Wei J. C., Preliminary report on the monitoring survey of sand flies in Wuxiang County, Shanxi Province. Chinese Journal of Parasitology and Parasitic Diseases 6 (1989).
158 Guan L. R. & Chai J. J., Kala-azar in the gravel desert zone of Xinjiang. Bulletin of Disease Control & Prevention (China) 40 (1989).
159 Yan Q. Y., Xu R. Y., Le Yuan Shang, Wang H. L. & Shi D. Y., Henan Province 1983 to 1988 kala-azar surveillance report. Bulletin of Disease Control & Prevention (China) 70 (1989).
160 Xu Y. X., Guan L. R., Wang G. & Chai J. J., Leishmaniasis in the Karamay region - I. Two cases of cutaneous leishmaniasis detected in Karamay, Xinjiang. Bulletin of Disease Control & Prevention (China) 70 (1989).
161 Guan L. R., Xu Y. X., Jia J. X. & Wang G., Experiments on artificial infection of Leishmania alexandrii with Leishmania dublin in different regions. Chinese Journal of Parasitology and Parasitic Diseases 152 (1988).
162 Jia J. X. et al., Experiments on the repellent effect of DETA, DMP, MRP and MRL on the Phlebotomus wui. Chinese Journal of Parasitology and Parasitic Diseases 153 (1988).
163 Ding S. D., Cai Y. L. & Wu S. S., Morphology of the larvae of the Sergentomyia bailyi and Sergentomyia zhengjian. Journal of Nanjing Medical University (Natural Sciences) 294 (1988).
164 Xiong G. H., Jin C. F. & Hong Y. M., A preliminary study on the species type and vertical distribution of the Phlebotomus chinensis in north Sichuan, Longnan. Bulletin of Disease Control & Prevention (China) 48 (1988).
165 Guan L. R., The biology of sand flies and sand flies and leishmaniasis. Bulletin of Disease Control & Prevention (China) 78 (1988).
166 Shu G. H., Discovery of a new genus and new species of the subfamily Sand fly in Gyunlian County, Sichuan. Journal of Preventive Medicine Information 124 (1988).
167 Xiong G. H., Jin C. F., Chai J. J. & Zuo X. P., The sand fly system of northern Xinjiang. Bulletin of Disease Control & Prevention (China) 67 (1988).
168 Jia J. X., Guan L. R., Xu Y. X., Wang G. & Hao K. F., Preliminary report on the repellent effect of DETA on Phlebotomus alexandri. Chinese Journal of Parasitology and Parasitic Diseases 67 (1988).
169 Yan Q. Y., Le Yuan Shang, Li D. F., Shi D. Y. & Yang Z. S., 1987 Sand fly Monitoring Survey in Henan Province. Journal of Pathogen Biology 20 (1988).
170 Xiong G. H. et al., A preliminary investigation on the relationship between the sand fly and kala-azar in Wen County, Longnan and Nanping, Sichuan. Bulletin of Disease Control & Prevention (China) 22 (1987).
171 Leng Y. J. & Zhang L. M., Two new species of Sergentomyia fukienensis (Diptera: Psychodidae). Acta Zootaxonomica Sinica 192 (1987).
172 Guan L. R., Xu Y. X., Zuo X. P. & Li B. S., Sand flies below sea level in the Turpan Basin, Xinjiang. Chinese Journal of Parasitology and Parasitic Diseases 46 (1987).
173 Guan L. R., Xu Y. X., Jia J. X., Wang G. & Zuo X. P., A study on the ecology of Phlebotomus alexandri in the Turpan Basin, Xinjiang. Bulletin of Disease Control & Prevention (China) 36 (1987).
174 Guan L. R., Xu Y. X., Mao Y. D. & Wang W., Sand flies and their transmission of kala-azar in different landscape zones of Aksu region, Xinjiang. Chinese Journal of Parasitology and Parasitic Diseases 11 (1986).
175 Jin C. F., Xiong G. H., Zuo X. P. & Chai J. J., The first account of the Sergentomyia arpaklensis in Xinjiang, China. Bulletin of Disease Control & Prevention (China) 134 (1986).
176 Shu G. H. & Sun Z. Z., Sand flies and their distribution in southern Sichuan. Journal of Medical Pest Control 39 (1986).

177 Zhang Z. G., Zhu Z. G. & Su Y. F., Epidemiological survey of kala-azar in Lueyang County. Shaanxi Medical Journal 15 (1985).
178 Lu Y. L. & Qiao Z. D., Investigation of sand flies in Jiexiu, Yangquan and Yuxian. Journal of Shanxi Medical University 3 (1985).
179 Lu Y. L. & Qiao Z. D., Survey on the systematics of sand flies in Xinzhou, Shanxi. Chinese Journal of Parasitology and Parasitic Diseases 7 (1985).
180 Ding S. D., Feng L. X., Huang L., Xia M. Y. & Zhou T., Scanning electron microscope observation of the larvae of Phlebotomus chinensis. Chinese Journal of Parasitology and Parasitic Diseases 62 (1985).
181 Ding S. D., Cai Y. L., Xu S., Feng L. X. & Yuan T. Z., Observations on the life history and some ecological habits of Phlebotomus kiangsuensis and Sergentomyia bailyi. Journal of Nanjing Medical University (Natural Sciences) 215 (1985).
182 Zhao E. M. et al., Epidemiological investigation of kala-azar in Bachu Reclamation Area, Xinjiang. Journal of Xinjiang Medical University 259 (1985).
183 Wu Z. X., Jiangsu Province kala-azar special report. Journal of Xuzhou Medical University 1 (1985).
184 Guan L. R., Xu Y. X., Li B. S. & Dong Q., Study on the transmission of kala-azar by Phlebotomus alexandri. Chinese Journal of Parasitology and Parasitic Diseases 7 (1985).
185 Di Wang G. & Yin Z. C., Phlebotomus chinensis and Phlebotomus kiangsuensis found for the first time in east Sichuan. Sichuan Journal of Zoology 16 (1985).
186 Xiong G. H., Zhao J. & Ge J. J., Self-fertility of the Phlebotomus chinensis. Acta Entomologica Sinica 70 (1985).
187 Cao H. X., Overview of leishmaniasis and sand fly research in Gansu Province. Journal of Lanzhou University (Medical Sciences) 33 (1984).
188 Lu Y. L., Qiao Z. D., Wang M. F. & Wu S. T., A survey of the sand fly system in representative counties of Shanxi Yanbei. Journal of Shanxi Medical University 18 (1984).
189 Ding S. D. & Feng L. X., Morphology of the larvae of Sergentomyia suni and Sergentomyia barraudi. Journal of Nanjing Medical University (Natural Sciences) 22 (1983).
190 Lu Y. L., Wu Y. X. & Zhang J., Survey on the distribution of sand flies in 11 counties and cities in Shanxi Province. Journal of Shanxi Medical University 7 (1983).
191 Guan L. R. et al., Investigation of Leishmania protozoa and its vectors in the giant gerbils and lizards in Ejina Banner, Inner Mongolia. Acta Academiae Medicinae Sinicae 261 (1982).
192 Guan L. R. et al., A study on the ecology of four species of sand flies in the desert of Ejinna Banner, Inner Mongolia. Journal of Lanzhou University (Medical Sciences) 140 (1982).
193 Ding S. D. & Feng L. X., Larval morphology of the Phlebotomus major wui and the sergentomyia minutus sinkiangensis subspecies. Acta Entomologica Sinica 264 (1982).
194 Wang C. T. & Jiang X. R., Analysis of the epidemiological survey of kala-azar from 1974 to 1980. NingXia Medical Journal 32 (1982).
195 Lu Y. L., Preliminary survey of the sand fly in west of Shanxi. Shanxi Medical Journal 35 (1982).
196 Deng Z. C. et al., Preliminary investigation of kala-azar and sand fly habit in Miyun County, Beijing Suburbs. Journal of Capital Medical University 14 (1982).
197 Xiong G. H. & Jin C. F., Sand fly system in Northwest China sand fly system. Entomotaxonomia 287 (1981).
198 Xiong G. H., Zhu X. Y. & Zhao J., The first discovery of autotrophic Phlebotomus chinensis Phlebotomus chinensis Newstead, 1916. Zoological Research 291 (1981).
199 Xiong G. H., Guan L. R. & Jin C. F., A new record of sand fly in northwest China and description of a new species of sand fly in the genus Sergentomyia. Acta Entomologica Sinica 430 (1981).
200 Lu Y. L., Sergentomyia suni was found for the first time in Shanxi Province. Journal of Shanxi Medical University 21 (1981).
201 Guan L. R., Jin C. F. & Xu Y. X., The new record of China's sand fly-Phlebotomus andrejevi. Entomotaxonomia 28 (1981).
202 Xiong G. H. et al., The discovery of an exotic lacewing genus in China. Acta Zootaxonomica Sinica 322 (1980).
203 Leng T. J., A study on the classification of Sergentomyia bailyi and its subspecies. Journal of Jinan University (Natural Science & Medicine Edition) 23 (1980).
204 Wen C. G., Sergentomyia bailyi found in Sichuan for the first time. Chinese Journal of Applied Entomology 129 (1980).
205 Guan L. R., Wang J., Liu P. Z. & Zhang Z. G., Ecology of the Phlebotomus chinensis in the mountainous areas of southern Gansu and the Loess Plateau of northern Shaanxi. Acta Entomologica Sinica 25 (1980).

206 Shu G. H., Wen X. M., Zhou H. X. & Peng Z. Z., Two species of sand flies found in Yibin area. Journal of Southwest Medical University 27 (1980).
207 Xiong G. H. et al., Ecological habits of Phlebotomus wui subspecies of desert sand fly in Xinjiang and its control. Acta Entomologica Sinica 428 (1979).
208 Leng Y. J., Liu Y. Q., Huang W. D. & Liu Z. X., New record of sand fly in Zhanjiang. Acta Zootaxonomica Sinica 189 (1979).
209 Wang C. T. et al., Observations on the ecological habits of Phlebotomus chinensis. Chinese Journal of Applied Entomology 161 (1979).
210 Wang H. L., Yan Q. Y. & Shi D. Y., A survey on the residual situation of sand fly in Henan Province. Henan Journal of Preventive Medicine 13 (1978).
211 Lu Y. L., Investigation and study of sand fly in Shanxi Province. Journal of Shanxi Medical University **5** 29 (1977).
212 Leng Y. J., A discussion on the taxonomy of Sergentomyia iyengari and its subspecies. Acta Entomologica Sinica 331 (1977).
213 Wang J. S., Gu Y. M. & Yuan T. Z., A new record and a new species of sand fly in Guizhou Province. Acta Entomologica Sinica 334 (1974).
214 Leng Y. J., A description of the sand fly species and a new sand fly species, Phlebotomus fanglianensis sp. nov. that continues to be found in Hainan Island. Acta Entomologica Sinica 118 (1964).
215 Xiong G. H., Wang J. & Guan L. R., Two species of sandflies of subgenus Paraphlebo. tomus found in North-West China. Acta Entomologica Sinica 141 (1964).
216 Wang J., Xiong G. H. & Liu P. Z., Ecological observation of Phlebotomus mongolensis in Gansu desert area. Acta Entomologica Sinica 679 (1963).
217 Xiong G. H., Wang J., De Hu Y. & Liu P. Z., Observations on the ecological habits of the Phlebotomus alexandri (Phlebotomus alexandri Sinton 1928). Acta Entomologica Sinica 458 (1963).
218 Wang Z. J. & Zhang R. S., A new species of sand fly in Sichuan - Phlebotomus tumenensis sp. n. Acta Entomologica Sinica 511 (1963).
219 Leng Y. J., Zhang Z. K. & Ji Y. Q., Liaoning Province Sand fly Investigation and Research II - Experiment on the extermination of sand fly by drugs. Chinese Journal of Applied Entomology 127 (1963).
220 Li F., Zhang Q. & Wang Y. W., Observations on the seasonal growth and decline of the Phlebotomus chinensis in Changchun (Abstract). Journal of Jilin University (Medicine Edition) 127 (1962).
221 Tang Z. Z. & Tang C. T., Description of a new species of Fujian Sand fly (Phlebotomus fukienensis sp. nov.). Journal of Fujian Normal University (Natural Science Edition) 161 (1959).
222 Wang J. S., Phlebotomus chinensis found in Guiyang for the first time. Acta Entomologica Sinica 490 (1959).
223 Cai H. Q., Yuan Y. J., He Z. X. & Chen C. C., Preliminary observations on the identification of Phlebotomus chinensis species. Acta Universitatis Medicinalis Anhui 181 (1959).
224 NA, A study on the ecology of sand fly in Gansu Province. Journal of Lanzhou University (Medical Sciences) 7 (1959).
225 NA, A study on the breeding habits of sand flies in Gansu Province. Journal of Lanzhou University (Medical Sciences) 18 (1959).
226 Hu C. R. & Zhang J. Q., Preliminary report on the survey of sand fly species in Xiangyang County, Hubei Province. Acta Entomologica Sinica 99 (1959).
227 Lu Y. L. & Zhang W. Z., A preliminary study on the species of sand fly and its ecology in Taiyuan City. Shanxi Medical Journal 1 (1958).
228 Xu Y. Q., First report of the discovery of sand fly in Shanghai. Acta Entomologica Sinica 496 (1957).
229 Leng Y. J. & Qin Y. T., A Preliminary Report on the Distribution of Sand fly Species and Genera in Liaoning Province. Chinese Journal of Applied Entomology 169 (1957).
230 Su S. Z. & Wang Y. Z., Supplementary report on the distribution survey of sand fly species in Kaifeng, Henan. Journal of Zhengzhou University (Medical Sciences) 23 (1957).
231 Gao J. M., Yao B. X., Wei B. X., Guo N. G. & Liu M. H., Preliminary observation of sand fly species and their seasonal distribution in Baoding. Acta Entomologica Sinica 529 (1956).
232 Wu Z. J., Wang Z. J., He K. Z. & Wang J., A study on the ecology of sand flies in East China from 1951 to 1953. Acta Entomologica Sinica 393 (1955).
233 Chen X. T. & Xu B. K., Report of the Guangdong sand fly and description of a new variant. Acta Entomologica Sinica 295 (1955).

234 He K. Z., Tan J. J. & Wu Z. J., A survey of the sand fly species in China--Nanjing and its vicinity and the new species "Sergentomyia nankingensis". Acta Entomologica Sinica 427 (1954).
235 Wu Z. J., A survey of the sand fly species in China VII - Two New Species of Sand fly in Shaanxi Province - Phlebotomus suni and Phlebotomus fupingensis. Acta Entomologica Sinica 287 (1954).
236 Xu C. B. et al., STUDIES ON THE HABITAT OF PHLEBOTOMUS CHINENSIS IN BEIJING SUBURBS. CHINESE MED J-PEKING **99** 920 (1986).
237 Meng K. Y., Chen Y. M., Ren G. Q., Yuan Y. F. & Luo W. J., Analysis of the current situation of kala-azar prevalence and control in Wudu District, Longnan City. Good Health for All **11** (2017).
238 Li K. L., Isolation and identification of symbiotic fungi of Phlebotomus chinensis in Sichuan and Henan provinces, China (2016).
239 Xu F., Taxonomy and molecular phylogeny of the sand fly based on morphological characteristics and mitochondrial Cytb gene, 2011.
240 Guan L. R., Research on the biological investigation of Phlebotomus in China. International Journal of Medical Parasitic Diseases **37** (2010).
241 Diwu J. X., Preliminary report on vector biology in the earthquake-stricken area of Longnan, Gansu province, China (2009).
242 Fu Q., Study on the temporal-spatial aggregation of human-derived kala-azar and its vector distribution in Kashgar region, 2007.
243 Zhang Y., Investigation of Phlebotomus chinensis in Jiuzhaigou County, Sichuan Province. International Journal of Medical Parasitic Diseases **34** (2007).
244 Zuo X. P., Maimaitijiang W. & Wei Y. S., Investigation report on the control of kala-azar in Kucha County from 1996 to 2006. Bulletin of Disease Control & Prevention (China) **22** (2007).
245 Gao C. H., Guan L. R. & Wang J. Y., Research progress on transmission mechanism of human leishmania and their vector sandfly species. International Journal of Medical Parasitic Diseases **34** (2007).
246 Wang X. Y., Lv J. J., Cao Z. X., He H. G. & Zheng Z. Y., Vector density monitoring and hazard assessment of Olympic competition venues and tourist attractions in Changping District. Chinese Journal of Vector Biology and Control **18** (2007).
247 Guan L. R., Gu D. A. & Wang J., Biology and control strategy of Phlebotomine sandfly. International Journal of Medical Parasitic Diseases **34** (2007).
248 Di S. Y., A survey of blood-sucking Diptera species in Hebei Province and analysis of their fauna, 2006.
249 Wang S. A., Lv J. J. & Mao Y. D., Investigation of kala-azar and its vector sand fly in Aksu area of Xinjiang. Chinese Journal of Epidemiology **06** (1985).
250 Wang H. L. & Yan Q. Y., The epidemiological surveillance of kala-azar after its eradication in Henan Province. Chinese Journal of Epidemiology **05** (1984).
251 Yang J. Q. et al., Studies on the ecology and elimination methods of sandfly. Chinese Journal of Epidemiology **05** (1984).
252 Hong J. W., Wu Z. J. & De Zhi Ma, A preliminary report on the blood-sucking habit of sand flies. National Medical Journal of China **42** (1956).
253 Lan Z. Y. & Gan S. S., Preliminary Report on the Ecological Habits of Sand flies in Qinan County, Gansu Province. Chinese Journal of Preventive Medicine **03** (1955).
254 Wu Z. J., The basic knowledge necessary for the study of sand flies. Chinese Journal of Internal Medicine **02** (1950).
255 YAO Y. T. & WU C. C., NOTES ON THE CHINESE SPECIES OF GENUS PHLEBOTOMUS. PART IV. DIAGNOSTIC TABLES FOR THE CHINESE SPECIES OF SANDFLIES WITH SOME REMARKS ON THEIR GEORGAPHICAL DISTRIBUTION. CHINESE MED J-PEKING **60** (1941).
256 Wang Q. Y., Study on the relationship between sand fly population and sand fly-transmitted virus in Yangquan City, Shanxi Province, 2021.
257 Xu X. Y., Isolation and identification of arboviruses in Wuxiang County, Shanxi Province, 2021.
258 Wang J., Isolation and identification of arboviruses in Shanxi Province, 2020.
259 Chen H. Y., DNA barcoding of common sand flies in China and transcriptome study of Phlebotomus chinensis, 2017.
260 Zhang L., Identification and molecular population genetic structure of the Phlebotomus chinensis in China, 2010.
261 Sun J. M., Numerical classification and DNA barcoding-based molecular phylogeny of sand flies, 2010.
262 Li K. L., Ma Y. J., Xu J. N. & Cao J., Isolation and identification of symbiotic bacteria of Phlebotomus chinensis, the vector of leishmaniasis in China (2016).

263 Leng Y. J., Two new sand fly species continue to be found in Hainan Island (1962).
264 Xiong G. H., Wang J. & Guan L. R., Discovery of two sandfly species, Phlebotomus alexandri and Phlebotomus caucasicus, in the subgenus Paraphebotomus in northwest China (1962).
265 Guo Q. F., Lv J. Y., Liu L. J., Chen H. & Meng W., Emergency epidemiological investigation of an animal associated with visceral leishmaniasis in young children in Henan Province. Sichuan Animal & Veterinary Sciences **49** 20 (2022).
266 Zhu J. L., Safeguarding life and health: kala-azar epidemic and prevention in Fuyang special zone (1951–1958). Journal of Jinzhou Medical University (Social Science Edition) **19** 23 (2021).
267 Gu Z. Q. et al., A bionomic investigation and analysis of sandflies, the vectors of kala-azar, in Sanmenxia, Henan Province, China. Chinese Journal of Vector Biology and Control **32** 590 (2021).
268 Li F. et al., Analysis of the environmental factors affecting leishmania infection in dogs in two counties of Gansu Province. Chinese Journal of Zoonoses **37** 763 (2021).
269 Li Y. X. et al., Epidemiological investigation on a new local case of visceral leishmaniasis in Luoyang City, Henan Province. Chinese Journal of Parasitology and Parasitic Diseases **39** 410 (2021).
270 Liao L. F., Adili S., Xu Y. M., Wu S. B. & Yan S. S., Investigation on natural host animals of desert type of kala-azar in Tarim Basin. Bulletin of Disease Control & Prevention (China) **36** 1 (2021).
271 Dai P. F. et al., The effectiveness comparative study of three different devices in trapping sandflies in different areas of Shanxi Province, China. Chinese Journal of Vector Biology and Control **31** 212 (2020).
272 Zhao X. & Leng Y. J., The research of Phlebotomine sandflies in Jilin and Heilongjiang provinces. Chinese Journal of Disease Control and Prevention 172 (1998).
273 Zhou Z. B. et al., Comparative performance of four different light-traps in a visceral leishmaniasis endemic focus in Shanxi Province. Journal of Practical Medical Techniques **28** 1387 (2021).
274 Leng Y. J., A preliminary survey of phlebotomine sandflies in limestone caves of Sichuan and Guizhou Provinces, south-west China, and description and discussion of a primitive new genus Chinius. Ann Trop Med Parasitol **81** 311 (1987).
275 Leng Y. J. & Zhang S. L., A study of phlebotomine sandflies (Diptera: Psychodidae) in Yunnan Province, China. Sergentomyia (Sergentomyia) wangi n. sp. PARASITE **6** 343 (1999).
276 Leng Y. J. & Zhang L. M., A study of phlebotomine sandflies (Diptera:Psychodidae) in Yunnan Province. III. Phlebotomus (Adlerius) fengi sp. nov. Ann Trop Med Parasitol **88** 523 (1994).
277 Zhang L. M., He M. S. & Ward R. D., A study of phlebotomine sandflies (Diptera:Psychodidae) in Yunnan Province. V. Phlebotomus (Larroussius) lengi sp. nov. Ann Trop Med Parasitol **88** 531 (1994).
278 Leng Y. J. & Zhang L. M., Check list and geographical distribution of phlebotomine sandflies in China. Ann Trop Med Parasitol **87** 83 (1993).
279 Leng Y. J. & Zhang L. M., Chinese phlebotomine sandflies of subgenus Adlerius nitzulescu, 1931 (Diptera: Psychodidae) and the identity of Phlebotomus sichuanensis Leng & Yin, 1983. Part I--Taxonomical study and geographical distribution. PARASITE **8** 3 (2001).
280 Zhang L. M. & Leng Y. J., Chinese phlebotomine sandflies of the subgenus Adlerius Nitzulescu, 1931 (Diptera: Psychodidae) and the identity of P. sichuanensis Leng & Yin, 1983. II--genotyping and numerical analysis. PARASITE **9** 287 (2002).
281 Guan L. R., Current status of kala-azar and vector control in China. B WORLD HEALTH ORGAN **69** 595 (1991).
282 Guan L. R., Yang Y. Q., Qu J. Q. & Shen W. X., Discovery and study of Leishmania turanica for the first time in China. B WORLD HEALTH ORGAN **73** 667 (1995).
283 Li K. et al., Diversity of bacteriome associated with Phlebotomus chinensis (Diptera: Psychodidae) sand flies in two wild populations from China. SCI REP-UK **6** 36406 (2016).
284 Chen H. et al., Ecological niches and blood sources of sand fly in an endemic focus of visceral leishmaniasis in Jiuzhaigou, Sichuan, China. INFECT DIS POVERTY **5** 33 (2016).
285 Zhang L. M. & Leng Y. J., Eighty-year research of phlebotomine sandflies (Diptera: Psychodidae) in China (1915–1995). II. Phlebotomine vectors of leishmaniasis in China. PARASITE **4** 299 (1997).
286 Wang J. et al., Emerging Sand Fly-Borne Phlebovirus in China. EMERG INFECT DIS **26** 2435 (2020).
287 Zhang L., Ma Y. & Xu J., Genetic differentiation between sandfly populations of Phlebotomus chinensis and Phlebotomus sichuanensis (Diptera: Psychodidae) in China inferred by microsatellites. PARASITE VECTOR **6** 115 (2013).

288 Wang J. et al., Isolation and Characterization of Wuxiang Virus from Sandflies Collected in Yangquan County, Shanxi Province, China. VECTOR-BORNE ZOONOT **21** 446 (2021).
289 Xu Z. et al., Isolation and Identification of a Novel Phlebovirus, Hedi Virus, from Sandflies Collected in China. VIRUSES-BASEL **13** (2021).
290 Wang Q. et al., Isolation and Identification of Sandfly-Borne Viruses from Sandflies Collected from June to August, 2019, in Yangquan County, China. VIRUSES-BASEL **14** (2022).
291 Chen H. M. et al., Leishmania infection and blood sources analysis in Phlebotomus chinensis (Diptera: Psychodidae) along extension region of the loess plateau, China. INFECT DIS POVERTY **9** 125 (2020).
292 Wei F. et al., Molecular detection and genetic diversity of Leishmania donovani in naturally infected Phlebotomus chinensi from southwestern China. VECTOR-BORNE ZOONOT **11** 849 (2011).
293 Guan L. R., Zhou Z. B., Jin C. F., Fu Q. & Chai J. J., Phlebotomine sand flies (Diptera: Psychodidae) transmitting visceral leishmaniasis and their geographical distribution in China: a review. INFECT DIS POVERTY **5** 15 (2016).
294 Wang Q. et al., Re-isolation of Wuxiang Virus from Wild Sandflies Collected from Yangquan County, China. VIROL SIN **36** 1177 (2021).
295 Chen Y. F. et al., Species identification and phylogenetic analysis of Leishmania isolated from patients, vectors and hares in the Xinjiang Autonomous Region, The People's Republic of China. PLOS NEGLECT TROP D **15** e10055 (2021).
296 Leng Y. J. & Lewis D. J., The subgenus Euphlebotomus (Diptera: Psychodidae) in China, with description of a new species, Phlebotomus yunshengensis. Ann Trop Med Parasitol **81** 305 (1987).
297 Leng Y. J. & Yin Z. C., The taxonomy of phlebotomine sandflies (Diptera: Psychodidae) of Sichuan Province, China, with descriptions of two species, Phlebotomus (Adlerius) sichuanensis sp. n. and Sergentomyia (Neophlebotomus) zhengjiani sp. n. Ann Trop Med Parasitol **77** 421 (1983).
298 Wang J. et al., Total RNA sequencing of Phlebotomus chinensis sandflies in China revealed viral, bacterial, and eukaryotic microbes potentially pathogenic to humans. EMERG MICROBES INFEC **11** 2080 (2022).
299 Xiong G. H., Jin C. F. & Guan L. R., Chinese Sandflies. (2016).
300 Lun Z. R. et al., Visceral Leishmaniasis in China: an Endemic Disease under Control. CLIN MICROBIOL REV **28** 987 (2015).
301 Xu X. et al., Wuxiang Virus Is a Virus Circulated Naturally in Wuxiang County, China. VECTOR-BORNE ZOONOT **21** 289 (2021).
