# Supplementary material for: Mapping the distribution of sandflies and sandfly-associated pathogens in China
Source: PLoS Negl Trop Dis. 2024 Jul 16;18(7):e0012291. doi: 10.1371/journal.pntd.0012291 (PMC11251628; doi:10.1371/journal.pntd.0012291)
Supplement: Attachment — Submitted filename: Response to Reviewers and editors.docx [file pntd.0012291.s003.docx]

PNTD-D-24-00442

**Mapping the distribution of sandflies and sandfly-associated pathogens in China**

Dear Dr. Luc E. Coffeng and Dr. Amy Morrison,

We appreciate your assistance in reviewing our manuscript and we also appreciate the

reviewer’s comments to improve our manuscript. We had addressed the reviewer’s

comments point by point. We hope the resubmission of the manuscript could be

accepted for publication in the **PLOS Neglected Tropical Diseases**.

Sincerely yours,

Wei Liu, M.D

State Key Laboratory of Pathogen and Biosecurity,

Academy of Military Medical Science

20 Dong-Da Street, Fengtai District, Beijing 100071, China

Tel: +86-10-63896082

Fax: +86-10-63896082

Email: lwbime@163.com or [liuwei@bmi.ac.cn](mailto:liuwei@bmi.ac.cn)

# Response to Reviewers’ Comments

## Reviewer #1:

### Major comments

This study comprehensively investigates the distribution of sandflies and sandfly-associated pathogens (SAPs) in China, as well as the incidence risk of human visceral leishmaniasis (VL). By analyzing literature data from 1940 to 2022 and surveillance data from 2014 to 2018, the research maps out the distribution of sandfly species and identifies ecological drivers using machine learning techniques. The findings indicate a significant underestimation of current sandfly distribution and VL risk, and predicted their future distribution and risk under three scenarios of climate and socioeconomic changes. The study highlights the need for enhanced surveillance and field investigations, particularly in regions where VL risk is projected to persist or increase amidst changing climate and socioeconomic scenarios. Overall, I think the paper offers valuable insights into an important area of neglected tropical diseases. I have several general comments:

**1.** The first paragraph of the Introduction is too general; please reshape the text to improve readability. Additionally, in the second paragraph of the Introduction, please introduce the situation of sandfly-associated pathogens and visceral leishmaniasis in China.

**Response:** We appreciate the reviewer’s valuable comments. We have restructured the first paragraph of the text introduction to enhance readability (page 6, lines 77–81, lines 83–84). Additionally, we have expanded the second paragraph to include an introduction to the situation concerning SAPs and VL in China (page 6, lines 88–91, lines 93–96).

**2.** In both Fig. 3 a-c, the provinces of Qinghai and Yunnan are not mentioned. I don’t quite understand why the VL annual incidence predicted by the model from 2014 to 2018 would lead to Qinghai and Yunnan becoming new endemic provinces. Please provide more detailed interpretation of this result. Furthermore, please clarify the meaning of the white color in the legend of Fig 3-4. For instance, in Fig 4 a-d, the white blocks are described as “0” or “no risk”. Is this because the reported data is actually zero, or because there is no data available? If the reported data for these provinces is zero, please clarify. If it is because there is no data available, the description is incorrect.

Response: We appreciate the reviewer’s valuable comments. Your observation is correct. In both Fig. 3 a-c, the provinces of Qinghai and Yunnan are not mentioned. However, as can be seen from Fig. 3c and Fig. 3d, the areas in Yunnan and Qinghai where VL is predicted to occur are close to the actual locations of vectors and VL in neighboring provinces. For example, in Qinghai province, which is adjacent to Gansu province where vectors and VL cases exist, due to the similar geographical location and various driving factor characteristics, our model is likely to predict VL cases in these provinces. As long as a specific area is predicted to have VL cases, we define the province where the specific area is located as an endemic province.

Regarding the legend of Fig. 3–4, in Fig. 3c, d, the white area represents provinces that are either actually or predicted to be non-endemic for VL, meaning there are no actual or predicted VL cases in these provinces. In Fig. 4a–c, the white block labeled “0–” signifies that the predicted incidence of VL in the area is between 0 and 0.071 per 100,000 population. In Fig. 4d–f, the white block labeled “no risk” indicates that the VL risk level has not changed compared to the previous period, and both periods are predicted to be “no risk”. The gray block labeled “0 (Stability)” represents areas where the risk level has not changed, but both periods are predicted to have VL risk. To distinguish between the two, areas where the risk level has not changed and remains “no risk” are separately represented in white. Detailed explanations have been added to the legend of Fig. 3–4 (page 18, lines 341–342; page 26, lines 455–457).

**3.** This is a good piece of work, but I believe the discussion section does not fully illustrate on the significance of this study. For example, in lines 525-526, the authors wrote, “Moreover, the expansion risk is greatest in the SSP585 scenario compared to the SSP126 and SSP245 scenarios.” This is not surprising because SSP585 inherently represents a high-emission scenario with a fossil-fuel intensive pathway. Currently, there is not enough discussion on the differences in results under different scenarios and the significance of conducting this analysis. I believe the authors should reorganize the discussion section to highlight the universal value of this study.

**Response:** We appreciate the reviewer’s valuable comments. We have supplemented the discussion section to elucidate the differences in results under various scenarios (page 31, lines 566–572) and the significance of conducting this analysis (pages 30–31, lines 563–566; page 31, lines 585–587), to underscore the universal value of this study.

### Specific Comments

**1.** Some of the keywords are too general; please use more appropriate terms.

**Response:** We appreciate the reviewer’s valuable comments. We have replaced or removed some overly general keywords and added some specific ones. (page 4, lines 57–58)

**2.** Lines 125-126: Please clearly state what the final selection of 8 bioclimatic variables is.

**Response:** We appreciate the reviewer’s valuable comments. In our revised manuscript, we have already stated the final selection of eight bioclimatic variables (page 9, line 154), and the detailed information about the selection of bioclimatic variables is presented in Appendix A: Tables S5.

**3.** Lines 340: Should it be six provinces instead of seven provinces?

**Response:** We appreciate the reviewer’s valuable comments. Corrected as suggested (page 20, line 373).

**4.** Lines 408-410: The description of the trend in VL incidence is not accurate. Due to the inconsistent trends in VL incidence under different Shared Socioeconomic Pathways scenarios and time periods, please clarify which specific scenario and time period the changes are based on.

**Response:** We appreciate the reviewer’s valuable comments. We have re-described the future trend in VL incidence based on specific scenarios and periods (page 24, line 442–447).

**5.** Lines 530-531: The description here is repetitive compared to lines 527-528.

**Response:** We appreciate the reviewer’s valuable comments. We have removed the repetitive content (page 31, lines 577–578).

**6.** Please reorganize the conclusion section. It seems too vague and does not highlight the key points of this study.

**Response:** We appreciate the reviewer’s valuable comments. We have reorganized the conclusion section to highlight the key points of our study (page 32, lines 598–614).

**7.** Please carefully review the format of the references, some of which is not consistent.

**Response:** We appreciate the reviewer’s valuable comments. We carefully reviewed the reference format and standardized the formatting (pages 35–39, lines 652–769).

## Reviewer #2:

**1.** The objectives and study design look fine. The clarity, appropriateness and size of the population under study are based largely on those from previously accepted publications. Sophisticated and advanced statistical programs were used for data analysis to support the conclusions. There are no ethical or regulatory concerns.

**Response:** Thanks for the reviewer’s positive comments.

**2.** There are ample and detailed descriptions presented according to analysis plan. For the most part, results are amply presented in some detail supported by figures with reasonable clarity.

**Response:** Thanks for the reviewer’s positive comments.

**3.** The data presented are in support of conclusions for the most part Some clear limitations of the analysis were listed and described. The potential applications of the approaches were stated with relevance to public health.

**Response:** Thanks for the reviewer’s positive comments.

**4.** The Abstract needs attention to make sure that it covers all the essential points of the work presented. For example, 6 known SAPs (sand fly-associated pathogens) were mentioned in the text, but only Leishmania appeared in the abstract.

**Response:** We appreciate the reviewer’s valuable comments. We have revised the ‘results’ section of the abstract and supplemented it with additional information about other SAPs (page 3, lines 39–44).

PNTD-D-24-00442

**Mapping the distribution of sandflies and sandfly-associated pathogens in China**

Dear Dr. Nithya Venkatesan,

We appreciate your assistance in reviewing our manuscript. We have made the requested modifications to our submission. We hope the resubmission of the manuscript could be accepted for publication in the **PLOS Neglected Tropical Diseases**.

Sincerely yours,

Wei Liu, M.D

State Key Laboratory of Pathogen and Biosecurity,

Academy of Military Medical Science

20 Dong-Da Street, Fengtai District, Beijing 100071, China

Tel: +86-10-63896082

Fax: +86-10-63896082

Email: lwbime@163.com or [liuwei@bmi.ac.cn](mailto:liuwei@bmi.ac.cn)

# Response to editor’s Comments

**1.** Please ensure that the CRediT author contributions listed for every co-author are completed accurately and in full. At this stage, "All Authors" require contributions. Please ensure that the full contributions of each author are acknowledged in the ""Add/Edit/Remove Authors"" section of our submission form.

**Response:** Thanks. We have carefully reviewed and followed the guidelines for CRediT author contributions. Each co-author’s contributions have been accurately and fully listed (page 33, lines 627–640). At this stage, contributions from “All Authors” have been included. We have ensured that the full contributions of each author are acknowledged in the “Add/Edit/Remove Authors” section of the submission form.

**2.** Please amend your detailed Financial Disclosure statement. This is published with the article, therefore should be completed in full sentences and contain the exact wording you wish to be published.

i). State the initials, alongside each funding source, of each author to receive each grant.

ii). State what role the funders took in the study. If the funders had no role in your study, please state: “The funders had no role in study design, data collection and analysis, decision to publish, or preparation of the manuscript.”

iii). If any authors received a salary from any of your funders, please state which authors and which funders.

**Response:** Thanks. We have updated our statement following your suggestions (page 33, lines 616–621).

**3.** Please provide an Author Summary. This should appear in your manuscript between the Abstract (if applicable) and the Introduction, and should be 150–200 words long. The aim should be to make your findings accessible to a wide audience that includes both scientists and non-scientists. Sample summaries can be found on our website under Submission Guidelines:

https://journals.plos.org/plosntds/s/submission-guidelines#loc-parts-of-a-submission

**Response:** Thanks. We have included an Author Summary in our manuscript (page 5, lines 60–71).

**4.** We noticed that you used "unpublished data" in the manuscript. We do not allow these references, as the PLOS data access policy requires that all data be either published with the manuscript or made available in a publicly accessible database. Please amend the supplementary material to include the referenced data or remove the references.

**Response:** Thank you for your instructions. Appendix B: Data S1 compiles data from all sources, including the unpublished data we described. We have indicated in the citations on page 8, line 141, and page 12, line 245, that the data are provided in Appendix B, Data S1. Furthermore, we have added a note in the title of the table in Appendix B: Data S1 stating, “These records summarize the distribution data of sandflies from all our collected sources.” This ensures that all data, including unpublished data, is accounted for and accessible.

**5.** Some material included in your submission may be copyrighted. According to PLOS’s copyright policy, authors who use figures or other material (e.g., graphics, clipart, maps) from another author or copyright holder must demonstrate or obtain permission to publish this material under the Creative Commons Attribution 4.0 International (CC BY 4.0) License used by PLOS journals. Please closely review the details of PLOS’s copyright requirements here: PLOS Licenses and Copyright. If you need to request permissions from a copyright holder, you may use PLOS's Copyright Content Permission form.

**Response:** Thank you for your instructions. We have provided a direct link to the base layer of the map in each figure that has potential copyright issues (page 16, lines 320–321; page 18, lines 345–346; page 26, lines 463–464; pages 40–45, lines 780–782, 790–792, 795–797, 800–802, 805–807, 811–812, 816–817, 875–877, 880–882, 885–887, 890–892, 895–897, 900–902), and have indicated the source of the map data in Appendix A: Text S1.
